# Comparing SARS-CoV-2 antigen-detection rapid diagnostic tests for COVID-19 self-testing/self-sampling with molecular and professional-use tests: a systematic review and meta-analysis

**DOI:** 10.1038/s41598-023-48892-x

**Published:** 2023-12-11

**Authors:** Stephan Katzenschlager, Lukas E. Brümmer, Stephani Schmitz, Hannah Tolle, Katharina Manten, Mary Gaeddert, Christian Erdmann, Andreas Lindner, Frank Tobian, Maurizio Grilli, Nira R. Pollock, Aurélien Macé, Berra Erkosar, Sergio Carmona, Stefano Ongarello, Cheryl C. Johnson, Jilian A. Sacks, Claudia M. Denkinger, Seda Yerlikaya

**Affiliations:** 1 Department of Anesthesiology, Medical Faculty Heidelberg, Heidelberg University, Heidelberg, Germany; 2 Division of Infectious Disease and Tropical Medicine, Center for Infectious Diseases, Heidelberg University Hospital, Im Neuenheimer Feld 324, 69120 Heidelberg, Germany; 3 German Center for Infection Research (DZIF), Partner Site Heidelberg University Hospital, Heidelberg, Germany; 4 Department of Developmental Biology, Erasmus Medical Center, Rotterdam, The Netherlands; 5 FH Muenster University of Applied Sciences, Muenster, Germany; 6 Charité Center for Global Health, Institute of International Health, Charité – Universitätsmedizin Berlin, Berlin, Germany; 7 University Medical Center Mannheim, Mannheim, Germany; 8 Department of Laboratory Medicine, Boston Children’s Hospital, Boston, MA USA; 9 FIND, Geneva, Switzerland; 10 Global HIV, Hepatitis and STIs Programmes, World Health Organization, Geneva, Switzerland; 11 Department of Epidemic and Pandemic Preparedness and Prevention, World Health Organization, Geneva, Switzerland

**Keywords:** Epidemiology, Infectious diseases

## Abstract

Self-testing is an effective tool to bridge the testing gap for several infectious diseases; however, its performance in detecting SARS-CoV-2 using antigen-detection rapid diagnostic tests (Ag-RDTs) has not been systematically reviewed. This study aimed to inform WHO guidelines by evaluating the accuracy of COVID-19 self-testing and self-sampling coupled with professional Ag-RDT conduct and interpretation. Articles on this topic were searched until November 7th, 2022. Concordance between self-testing/self-sampling and fully professional-use Ag-RDTs was assessed using Cohen’s kappa. Bivariate meta-analysis yielded pooled performance estimates. Quality and certainty of evidence were evaluated using QUADAS-2 and GRADE tools. Among 43 studies included, twelve reported on self-testing, and 31 assessed self-sampling only. Around 49.6% showed low risk of bias. Overall concordance with professional-use Ag-RDTs was high (kappa 0.91 [95% confidence interval (CI) 0.88–0.94]). Comparing self-testing/self-sampling to molecular testing, the pooled sensitivity and specificity were 70.5% (95% CI 64.3–76.0) and 99.4% (95% CI 99.1–99.6), respectively. Higher sensitivity (i.e., 93.6% [95% CI 90.4–96.8] for Ct < 25) was estimated in subgroups with higher viral loads using Ct values as a proxy. Despite high heterogeneity among studies, COVID-19 self-testing/self-sampling exhibits high concordance with professional-use Ag-RDTs. This suggests that self-testing/self-sampling can be offered as part of COVID-19 testing strategies.

Trial registration: PROSPERO: CRD42021250706.

## Introduction

Self-testing allows individuals to collect their own sample, conduct the diagnostic test, and interpret the result. A growing body of evidence has shown self-testing with simple antigen-detection rapid diagnostic tests (Ag-RDTs) to be feasible, acceptable, and accurate^1^. Over the last decade, particularly for HIV and Hepatitis C, self-testing using lateral flow assays have shown high agreement and increased testing uptake in comparison to professional testing, as well as a low failure rate^2–5^. As a result, the World Health Organization (WHO) recommended self-testing for HIV in 2016 and for Hepatitis C in 2021^6,7^.

With the emergence of the COVID-19 pandemic, Ag-RDTs for SARS-CoV-2 became widely available. While less accurate compared to the gold standard nucleic acid amplification tests, Ag-RDTs enabled easy-to-use and rapid point-of-care (POC) testing^8^. This resulted in the WHO recommendation of SARS-CoV-2 Ag-RDTs for various use cases, including primary case detection and contact tracing^9^. Further, a sensitivity target of ≥ 80% has been recommended for Ag-RDTs^10^. However, the limited number of professional test operators hampered scale-up of and timely access to testing.

Building on the self-testing experiences for HIV and Hepatitis C, self-sampling coupled with professional Ag-RDT test conduct and interpretation (henceforth named self-sampling) as well as self-testing for COVID-19 was explored^11–13^. However, to date, no systematic review focusing solely on the performance of Ag-RDT self-testing and/or self-sampling has been performed. To address this knowledge gap and inform WHO guideline development, we conducted a systematic review and meta-analysis to (1) assess the concordance between self-testing and/or self-sampling and professional testing using commercially available Ag-RDTs for SARS-CoV-2 and (2) assess the accuracy of self-testing and/or self-sampling for COVID-19 using commercially available Ag-RDTs against reverse transcription polymerase chain reaction (RT-PCR) performed on self-collected or professionally-collected samples.

## Methods

The methods were adapted from a living systematic review our group had previously published^8,14^. The systematic review protocol (Supplement, S1 Text Study Protocol) is registered on PROSPERO (CRD42021250706). We followed the Preferred Items for Systematic Reviews and Meta-analysis (PRISMA) guideline to report our findings (Supplement, PRISMA Checklist)^15^.

### Search strategy

We searched the databases MEDLINE (via PubMed), Web of Science, medRxiv, and bioRxiv (via Europe PMC), using search terms developed with an experienced medical librarian (MGr) using combinations of subject headings (when applicable) and text words for the concepts of the search question. The main search terms were “Severe Acute Respiratory Syndrome Coronavirus 2,” “COVID-19,” “Betacoronavirus,” “Coronavirus,” and “Point of Care Testing” and checked against an expert-assembled list of relevant papers. The full list of search terms is available in the supplementary material (Supplement Text 2Search Strategy). Furthermore, we looked for relevant studies on the FIND website (https://www.finddx.org/sarscov2-eval-antigen/). We conducted the search without applying any language, age, or geographic restrictions from inception up until November 7th, 2022.

### Eligibility criteria

We included studies evaluating the accuracy of self-testing and/or self-sampling using commercially available Ag-RDTs to establish a diagnosis of SARS-CoV-2 infection against RT-PCR as the reference standard. In studies assessing self-sampling, the Ag-RDT performance (including readout and interpretation) was conducted by a professional. Sampling conducted or assisted by caregivers was included as self-sampling. RT-PCR samples were eligible if they were either self-collected or professionally-collected without a restriction on sample type (henceforth referred to as ‘RT-PCR’).

We included all studies reporting on any population, irrespective of age, symptom presence, or study location. We considered cohort studies, nested cohort studies, case–control, cross-sectional studies, and randomized controlled trials (RCTs). We included both peer-reviewed publications and preprints. We excluded studies in which persons underwent testing for the purposes of monitoring or ending quarantine. In addition, publications with a sample size under ten were excluded to minimize bias in clinical performance estimates.

### Assessment of methodological quality

The quality of clinical accuracy studies was assessed by applying the quality assessment of studies of diagnostic accuracy (QUADAS-2) tool, which was adjusted to the needs of this review^16^. Details can be found in the supplementary material (Supplement Text 3 QUADAS).

### Assessment of certainty of evidence (CoE)

We defined three individual outcomes for this review: (1) concordance between self-testing/self-sampling coupled with professional Ag-RDT conduct and interpretation and fully professional-use Ag-RDTs, calculating Cohen’s kappa as well as positive percentage agreement (PPA), negative percentage agreement (NPA), and overall percentage agreement (OPA), (2) sensitivity, and (3) specificity against RT-PCR performed on a self-collected or professionally-collected sample as reference.

Certainty of evidence (CoE) was assessed following the GRADE guidelines for each individual outcome^17^. After rating the respective study type (e.g., RCT or observational trial), each outcome was independently evaluated according to five categories: study design, risk of bias (RoB), inconsistency, indirectness, and imprecision.

### Assessment of independence from manufacturers

We examined whether a study received financial support from a test manufacturer (including free provision of Ag-RDTs), whether any study authors were affiliated with the manufacturer, and whether a respective conflict of interest was declared. If at least one of these conditions was met, the study was deemed as not independent from the test manufacturer; otherwise, it was considered as independent.

### Statistical analysis and data synthesis

We extracted data from eligible studies using a standardized data extraction form. Wherever possible we recalculated performance estimates based on the extracted data or contacted authors to provide additional information on concordance between self-tested and professionally tested Ag-RDTs. The final data set used is accessible under https://doi.org/10.11588/data/P9JEPG.

We calculated Cohen’s kappa as a measure of concordance, its variance, and 95% confidence intervals (CIs) for comparison of results with fully professional-use Ag-RDTs. If four or more studies with at least 20 positive samples were available, we conducted a meta-analysis of Cohen’s kappa using the “metafor" package version 3.4-0 in R^18^. PPA, NPA, and OPA were additionally calculated using the following formulas when comparing self-testing/self-sampling with professional-use Ag-RDTs^19^:

**Table:** 

	Professional Ag-RDT Positive	Professional Ag-RDT Negative
Self-testing/self-sampling positive	a	b
Self-test/self-sampling negative	c	d

PPA = $$\frac{a}{(a+c)}*100\%$$;

NPA = $$\frac{d}{(b+d)}*100\%$$;

OPA = $$\frac{(a+d)}{(a+b+c+d)}*100\%$$;

We derived the estimates for sensitivity and specificity against RT-PCR and performed meta-analysis using a bivariate model when at least four data sets, each with at least 20 positive samples, were available (meta-analysis was implemented with “reitsma” command from the R package “mada,” version 0.5.11). If less than four studies were available for an outcome, only a descriptive analysis was performed, and accuracy ranges were reported. Univariate random-effects inverse variance meta-analysis was performed (using the “metaprop” and “metagen” commands from the R package “meta,” version 5.5–0) for the pooled sensitivity analysis per Ct values. We predefined subgroups for meta-analysis based on the following characteristics: Ct value range (< 20, < 25, < 30, ≥ 20, ≥ 25, ≥ 30), sampling and testing procedure in accordance with manufacturer and/or study team instructions (‘IFU-conforming’ versus ‘not IFU-conforming’), patient age (‘ < 18 years’ vs. ‘ ≥ 18 years’), presence of symptoms (‘symptomatic’ versus ‘asymptomatic’), and duration of symptoms (‘DoS ≤ 7 days’ vs. ‘DoS > 7 days’).

To make the most of the heterogeneous data available, the cutoffs for the Ct value groups were relaxed by up to three points within each range (e.g., Ct value range group < 20 can include studies with Ct values ≤ 17 to ≤ 23). For the same reason, when categorizing by age, the age group < 18 years (children) included samples from persons whose age was reported as < 16 or < 18 years, whereas the age group ≥ 18 years included samples from persons whose age was reported as ≥ 16 years or ≥ 18. Additionally, samples from the anterior nares (AN) and nasal mid-turbinate (NMT) were summarized as AN. IFU-conformity was judged based on the study team’s information. As self-testing was an off-label use at that time for some Ag-RDTs, following the study team’s instructions was defined as IFU-conforming. Observed sampling and testing were defined when a professional watched the testing procedure without intervening. Predominant variants of concern (VoC) for each study were analyzed using the online tool CoVariants^20^ with respect to the stated study period. The respective VoCs were extracted according the current WHO listing^21^. As Ag-RDTs should be used in settings where RT-PCR testing is limited^22^, an exploratory analysis comparing middle-income countries (MIC) and high-income countries (HIC) was also performed.

Heterogeneity was interpreted visually in forest plots. Further, we performed the Deeks test for funnel-plot asymmetry as recommended for diagnostic test accuracy meta-analyses to investigate small study effects^23^ (using the “midas” command in Stata, version 15); a *p*-value < 0.10 for the slope coefficient indicates significant asymmetry. Remaining analyses were performed using R 4.2.1 (R Foundation for Statistical Computing, Vienna, Austria).

### Sensitivity analysis

Three types of sensitivity analyses were planned: concordance and estimation of performance (sensitivity, specificity) of self-testing and/or self-sampling compared to RT-PCR excluding case–control studies, preprints, and manufacturer-dependent studies. We compared the results of the respective sensitivity analysis against the overall results to assess the potential bias.

## Results

**Figure 1:**
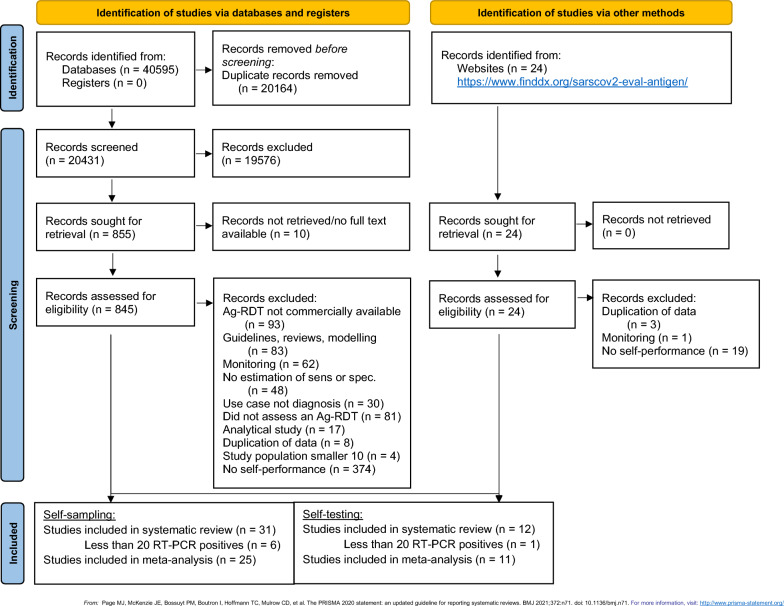
PRISMA flow diagram. Adapted from Page et al.^15^. Abbreviations: Ag-RDT = antigen rapid diagnostic test; RT-PCR = reverse transcription polymerase chain reaction; sens = sensitivity; spec = specificity.

Our search strategy yielded a total of 20,431 titles after removal of duplicates. Twelve studies^11,24–34^ incorporating 28 data sets on self-testing (27,506 samples) and 31 studies^12,13,35–63^ incorporating 37 data sets on self-sampling (31,792 number of samples) were found to be eligible for inclusion in the review (Fig. 1). One study was analyzed as self-sampling because it was unclear whether or not self-testing was performed^63^.

### Methodological quality of all included studies

**Figure 2:**
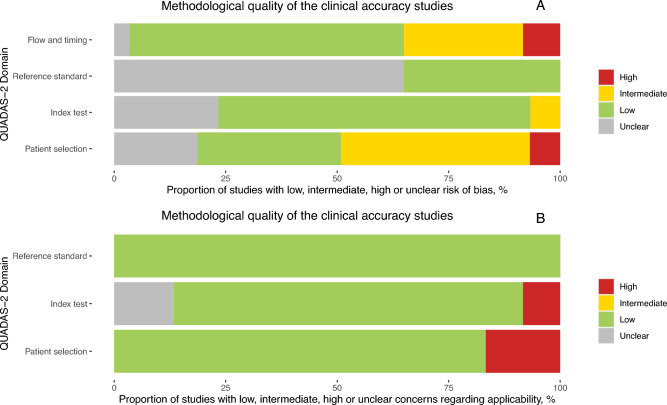
(**A**) QUADAS assessment for risk of bias and (**B**) applicability.

The included studies were assessed to be of high applicability overall and variable bias (Fig. 2A).

Low risk of bias was observed in 41 out of 65 datasets (63.1%), when assessing the timing of the index test, the inclusion of participants, and whether the same reference standard was used throughout the study. However, in only 40.0% of the studies were the results of the reference standard (PCR) interpreted without knowledge of the index test results; this was unclear for the remaining 60.0%. For 67.7% of the studies, the conduct and interpretation of the index test was of low concern because the Ag-RDT results were interpreted without knowledge of results of the reference standard. Only 33.8% of the studies had a representative study population, avoiding inappropriate exclusions or a case–control design thereby resulting in low risk of bias. Out of the remaining studies, the risk of bias for patient selection remained unclear for 16.9%, and 6.2% had high risk of bias and 43.1% had an intermediate risk of bias. Applicability was deemed to be of low concern in 86.2% of the studies across all domains since the methods (i.e., patient selection, index test conduct, reference standard choice) in the respective studies matched our research question (Fig. 2B; with further details in Supplementary Fig. 1). Potential conflict of interest due to financial support from or employment by the test manufacturer was present in 17 studies (34.7%) ^26,28,32,38,39,47,51,55,56,58,59,61–63^. In studies focusing on self-sampling, 30 out of 36 datasets reported IFU-conform conduct of the test, even though sampling was explicitly observed in only 22 datasets (61.1%). For studies evaluating self-testing, 26 datasets stated IFU-conformity, while for the remaining two datasets it was unclear.

With a p value of 0.31 and a roughly symmetrical funnel plot, analysis of small study effects—which may indicate publication bias—produced no significant evidence for such effects (Supplement, S2 Figure Funnel Plot).

### Study description

Most of the studies included in the review were conducted in high-income countries (HIC): the USA (n = 10), Germany (n = 7), the Netherlands (n = 6), UK, and Canada (n = 2, each), as well as Greece, Denmark, Japan, France, Belgium, Austria, France, Korea, and Hong Kong (n = 1, each). On the contrary, seven studies were conducted in middle-income countries (MIC): India (n = 3), Brazil, Morocco, Malaysia, and China (n = 1, each)^64^. No studies were performed in low-income countries. Considering the study participant’s level of education, in two studies reporting on self-testing, the majority of participants (59.6% and 98.1%) had at least a high school degree^11,24^. Out of the 17 studies reporting on self-sampling, one study stated that 52.5% of participants had a higher education degree^35^. Another study included only high school students (78.6%) or teachers (21.4%)^46^, while two other studies included only college students ^36,43^. The remaining studies provided no information on the participants’ educational backgrounds. Participants had prior medical training (i.e., health care worker) in three self-sampling datasets (2506 samples, 9.1%)^12,35^. Participants were lay people without any medical training for six datasets totaling 5023 samples, but for the other datasets, it remained unclear. Information on the participants' professional backgrounds and prior testing experiences was only reported in one self-testing study^10^. Out of the 144 participants in this study, 12 (8.3%) had prior medical training, 66 (45.8%) had undergone SARS-CoV-2 testing in the past, and four (2.8%) had performed at-home COVID-19 testing.

Most of the self-sampling data (32 datasets; 88.9%) were collected at testing or clinical sites, while for others no information was available. The sampling process was observed in 17 of the self-sampling studies (22 datasets), totaling 19,280 samples (60.6%)^12,13,37–41,43,46,48,49,51,52,54,58,61^, whereas sampling was not observed in four studies (4 datasets; 10.8%)^35,36,47,59^. For the remaining ten studies (10 datasets; 27.0%), it was unclear whether the sampling was observed or not^42,44,45,50,53,55–57,60,62^. Overall, 78.6% of the self-testing studies were carried out at a testing site, and the testing procedure was observed (without providing instructions) by the study team in three studies (1083 samples; 2.9%)^11,28,32^.

A total of 27,506 samples were evaluated in the self-testing studies. With 13,166 individuals presenting with symptoms suggestive of a SARS-CoV-2 infection, while 10,103 persons did not show any symptoms at the time of testing. For the rest, the authors did not specify the participants’ symptom status. A total of 31,069 individuals participated in the self-sampling studies, of whom 6325 had symptoms, 20,569 were asymptomatic, and 4175 had unclear symptom status.

The most used Ag-RDTs across all studies were the BinaxNow nasal test by Abbott (USA, henceforth called BinaxNow) and the Standard Q nasal test by SD Biosensor (South Korea; distributed in Europe by Roche, Germany; henceforth called Standard Q nasal), with six datasets each. The BD Veritor lateral flow test for Rapid Detection of SARS-CoV-2 (Becton, Dickinson and Company, MD, US; henceforth called BD Veritor), the CLINITEST Rapid COVID-19 Antigen Test (Siemens Healthineers, Germany; henceforth called CLINITEST), and the Rapid SARS-CoV-2 Antigen Test (MP Biomedicals, CA, US; henceforth called MP Bio) were used in three datasets each.

Most self-samples for antigen testing were taken from the anterior nares (‘AN’; 28 datasets, 77.7%). The remaining datasets made use of either combined oropharyngeal/anterior nasal (OP/AN) (2 datasets, 5.6%), saliva (2 dataset, 5.6%), a combination of the above (AN/saliva, 1 dataset, 2.8%), or OP (3 datasets, 8.3%) samples. Similarly, many self-testing datasets used AN sample (20 datasets, 71.4%); whereas OP/AN and saliva accounted for 4 datasets (14.3%) each. The following samples were used for RT-PCR testing: AN (15 datasets, 23.0%), nasopharyngeal (NP) (21 datasets, 32.3%), NP/OP (13 datasets, 20.0%), OP (7 datasets, 10.7%), OP/AN (5 datasets, 7.7%), or saliva (3 dataset, 4.6%). In one dataset (1.5%) the sampling type was not stated by the authors.

The RT-PCR and Ag-RDT analyses were conducted on the same sample type across 20 self-sampling datasets^31,36,39–48,51,54,55,59^. Self-collected samples were used for RT-PCR in 14 of those datasets^36,40,41,43,46–48,51,54,55^. In all self-testing studies, RT-PCR samples were collected by a professional (Table 1).

**Table 1:** Overview of possible sampling combinations in self-testing and self-sampling studies.

Sample type self-testing/sampling Ag-RDT	Sample type RT-PCR	n datasets (%)	Sample type professional Ag-RDT	n datasets (%)
AN	NP/OP	11 (17%)	–	
	NP	15 (23%)	NP	4 (57%)
	AN	15 (23%)	AN	2 (29%)
	OP	7 (11%)	OP	1 (14%)
	OP/nasal	2 (3%)	–	
AN/saliva	saliva	1 (2%)	–	
OP/nasal	OP/nasal	3 (5%)	–	
	NP	2 (3%)	–	
	NP/OP	1 (2%)	–	
OP	NP	1 (2%)	–	
Saliva	Saliva	2 (3%)	–	
	NP	3 (5%)	–	
	NP/OP	1 (2%)	–	

Percentages might not add up to 100% as they are rounded.

*AN* Anterior nasal, *OP* Oropharyngeal, *NP* Nasopharyngeal, *Ag-RDT* Antigen detection rapid diagnostic test, *RT-PCR* Reverse transcription polymerase chain reaction; In one study, RT-PCR sample type was unclear.

Two self-testing and one self-sampling studies provided additional instructional videos^24,29,45^. Regarding self-testing studies, four studies provided study-specific test instructions since no manufacturer instructions for self-testing were available at the time^11,24,25,29^.

Table 2 provides further information on each of the studies included in the review.

**Table 2:** a Clinical accuracy data for self-sampled Ag-RDTs. b Clinical accuracy data for self-testing Ag-RDTs.

Study	Test assessed	Country	Type of location	Study population	Screening criteria	Sample type	Sensitivity (95% CI)	Specificity (95% CI)
Harris^12^	Sofia	USA	testing site	adults	sympt., HRC	AN	82.3% (77.5^#^ to 86.4^#^)	98.8%^#^ (97.5^#^ to 99.5^#^)
Harris^12^	Sofia	USA	testing site	adults	asympt	AN	31.6% (0.0^#^ to 24.7^#^)	100% (99.8^#^ to 100)
Lindner^13^	Standard Q	Germany	testing site	adults	sympt	AN*	74.4% (57.9^#^ to 87.0^#^)	99.2% (97.1 to 99.9^#^
Tinker^43^	BinaxNow	USA	testing site	adults	asympt	AN*	20.0% (9.1^#^ to 35.6^#^)	100% (99.8^#^ to 100#)
Tanimoto^44^	Lumipulse	Japan	unclear	unclear	unclear	saliva	61.8% (47.7^#^ to 74.6^#^)	100% (94.1 to 100)
Mak^45^	Standard Q	Hong Kong	testing site	unclear	HRC	OP/AN*	100% (15.8^#^ to 100)	100% (90.7^#^ to 100)
Blanchard^70^	Panbio nasal	Canada	testing site	adults, children	sympt	AN*	78.6% (49.2^#^ to 95.3^#^)	100% (98.7^#^ to 100)
Harmon^47^	E25Bio	USA	testing site	adults	sympt., asympt	AN	92.3% (64.0^#^ to 99.8^#^)	99.6% (97.7^#^ to 100)
Ford^48^	BinaxNow	USA	testing site	children	sympt., HRC, asympt	AN*	71.4% (53.7 to 85.4)	100% (98.0 to 100)
Ford^48^	BinaxNow	USA	testing site	adults	sympt., HRC, asympt	AN*	80.9% (75.9 to 85.3)	99.9% (99.5 to 100)
Klein ^49^	Panbio nasal	Germany	testing site	adults	sympt., HRC	AN	86.4% (72.6^#^ to 94.8^#^)	99.2% (97.0 to 99.9^#^)
Nikolai^35^	Standard Q	Germany	clinical	adults	sympt	AN	91.2% (76.3^#^ to 98.1^#^)	98.4% (91.3^#^ to 100^#^)
Okoye^36^	BinaxNow	USA	testing site	adults	asympt	AN*	53.3% (37.9^#^ to 68.3^#^)	100% (99.9 to 100)
Krüger^37^	LumiraDx	Germany	testing site	adults	sympt., HRC	AN	82.2% (75.0^#^ to 88.0^#^)	99.3% (98.3 to 99.7)
Osmanodja^38^	Dräger	Germany	both	adults	sympt., asympt	AN	88.6% (78.7 to 94.9)	99.7% (98.2 to 100)
Chiu^39^	Indicaid	USA	clinical	adults, children	Sympt	AN	82.7% (72.2^#^ to 90.4^#^)	96.4% (93.4 to 98.2^#^)
García-Fiñana^40^	Innova	UK	testing site	adults	Asympt	OP/AN	40.0% (28.5 to 52.4)	99.9% (99.8 to 99.9)
Shah^41^	BinaxNow	USA	testing site	adults, children	sympt, HRC, asympt	AN	81.4% (76.8 to 85.5)	99.6% (99.2 to 99.8)
Frediani^42^	BinaxNow	USA	unclear	adults, children	unclear	AN	56.2%^#^ (29.9^#^ to 80.2^#^)	100% (87.7^#^ to 100)
Tinker^43^	BinaxNow	USA	testing site	adult	asympt	AN*	20.0 (9.1^#^ to 35.6^#^)	100 (99.8^#^ to 100^#^)
Tanimoto^44^	Lumipulse	Japan	unclear	unclear	unclear	saliva	61.8 (47.7^#^ to 74.6^#^)	100 (94.1 to 100)
Mak^45^	Standard Q	Hong Kong	testing site	unclear	HRC	OP/nasal	100 (15.8^#^ to 100)	100 (90.7^#^ to 100)
Blanchard^46^	Panbio nasal	Canada	testing site	adult, children	Sympt	AN*	78.6 (49.2^#^ to 95.3^#^)	100 (98.7^#^ to 100)
Harmon^47^	E25Bio	USA	testing site	adult	sympt., asympt	AN*	92.3 (64.0^#^ to 99.8^#^)	99.6 (97.7^#^ to 100)
Ford^48^	BinaxNow	USA	testing site	children	sympt, HRC, asympt	AN*	71.4 (53.7 to 85.4)	100 (98.0 to 100)
Ford^48^	BinaxNow	USA	testing site	adult	sympt, HRC, asympt	AN*	80.9 (75.9 to 85.3)	99.9 (99.5 to 100)
Ahmed^50^	ProDetect	Malaysia	unclear	adult, children	sympt, HRC,	AN	96.1^#^ (86.5^#^ to 99.5^#^)	98.0 (89.1^#^ to 99.9^#^)
Cardoso^51^	Wondfo	Brazil	testing site	unclear	sympt	AN*	73.0 (64.7^#^ to 80.2^#^)	98.6 (95.2 to 99.8^#^)
Chen^52^	Labnovation	China	clinical	adult	unclear	AN	70.4^#^ (49.8^#^ to 86.2^#^)	100^#^ (29.2^#^ to 100^#^)
Chen^52^	Labnovation	China	clinical	adult	unclear	AN	81.4^#^ (66.6^#^ to 91.6^#^)	64.0^#^ (42.5^#^ to 82.0^#^)
Gagnaire^53^	Biospeedia	France	testing site	adult, children	sympt, HRC, asympt	AN/saliva	59.4 (51.5 to 67.0)	99.8 (99.7^#^ to 99.9)
Goodall^54^	Panbio	Canada	testing site	unclear	asympt	AN*	64.5 (51.3^#^ to 76.3^#^)	100 (99.5^#^ to 100^#^)
Goodall^54^	Panbio	Canada	testing site	unclear	asympt	TN*	64.5 (51.3^#^ to 76.3^#^)	100 (99.5^#^ to 100^#^)
Goodall^54^	Panbio	Canada	testing site	unclear	asympt	AN*	68.4 (51.3^#^ to 82.5^#^)	100 (99.2^#^ to 100^#^)
Goodall^54^	Panbio	Canada	testing site	unclear	asympt	TN*	81.6 (65.7^#^ to 92.3^#^)	100 (99.2^#^ to 100^#^)
Igloi^55^	Standard Q	Netherlands	testing site	adult	sympt., HRC	saliva*	66.1 (52.9 to 77.6)	99.6 (98.8 to 99.9
Mane^56^	Coviself	India	testing site	adult	sympt., HRC	OP	54.2^#^ (39.2^#^ to 68.6^#^)	96.9^#^ (92.9^#^ to 99.0^#^)
Rangaiah^57^	Coviself	India	unclear	unclear	unclear	AN	61.5 (50.7 to 71.5)	100 (97.4 to 100)
Robinson^58^	BD Veritor nasal	USA	testing site	unclear	Sympt., HRC,	AN	-	-
Savage^59^	Covios	UK	testing site	adult	sympt	AN	90.5 (83.9 to 97.2)	99.4 (98.3 to 100)
Shin^60^	Standard Q	Korea	clinical	unclear	sympt., asympt	AN	94.9 (87.5 to 98.6)	100 (98.3 to 100)
Sukumaran^61^	AG-Q	India	clinical	unclear	unclear	AN	77.9 (67.7 to 86.1)	100 (94.4 to 100)
Tsao^63^	BinaxNow	USA	testing site	adult	sympt., asympt	AN	63.0 (50.9^#^ to 74.0^#^)	99.8 (99.1^#^ to 100)
Wölfl-Duchek^62^	Medomics	Austria	clinical	adult	sympt., asympt	AN	63.0 (47.5 to 76.8)	100 (91.0^#^ to 100)

Study	Test assessed	Country	Type of location	Study population	Screening criteria	Sample type	Sensitivity (95%CI)	Specificity (95%CI)
Lindner^11^	Standard Q	Germany	Clinical	Adults	sympt	AN	82.5% (67.2^#^ to 92.7^#^)	100% (96.5 to 100)
Stohr^22^	BD Veritor	Netherlands	Testing site	Adults	sympt., asympt	AN	48.9% (41.3^#^ to 56.5^#^)	99.9% (99.5 to 100)
Stohr^22^	Standard Q	Netherlands	Testing site	Adults	sympt., asympt	AN	61.5% (54.2^#^ to 68.4^#^)	99.7% (99.3 to 99.9)
De Meyer^25^	V-Chek	Belgium	Testing site	Adult, children	unclear	saliva	7.7 (0.2^#^ to 36.0^#^)	100 (90.5^#^ to 100^#^)
De Meyer^25^	Whistling	Belgium	Testing site	Adult, children	unclear	saliva	9.1 (3.0^#^ to 20.0^#^)	100 (92.5^#^ to 100^#^)
Diawara^26^	PCL	Morocco	Unclear	Adult, children	unclear	saliva	90.1 (80.7 to 95.9)	99.6 (97.9 to 99.9)
Diawara^26^	PCL	Morocco	Unclear	Adult, children	unclear	AN	91.4^#^ (82.3^#^ to 96.8^#^)	100 (98.5 to 100)
Iftner^27^	Anbio	Germany	Testing site	Adult	asympt	AN	–	99.8^#^ (98.8^#^ to 100^#^)
Iftner^27^	Clungene	Germany	Testing site	Adult	asympt	AN	–	97.9^#^ (96.2^#^ to 99.0^#^)
Iftner^27^	Hotgen	Germany	Testing site	Adult	asympt	AN	–	99.8^#^ (98.8^#^ to 100^#^)
Iftner^27^	Mexacare	Germany	Testing site	Adult	asympt	AN	–	99.8^#^ (98.8^#^ to 100^#^)
Leventopoulos^28^	Boson	Greece	Testing site	Adult, children	sympt., asympt	AN	98.2 (96.7 to 99.6)	100 (99.9 to 100)
Møller^29^	DNA Diagnostics	Denmark	Testing site	Adult	sympt, HRC, asympt	AN	65.7 (49.2 to 79.2)	100 (99.0 to 100)
Møller^29^	Hangzhou	Denmark	Testing site	Adult	sympt, HRC, asympt	AN	62.1 (50.1 to 72.9)	100 (98.9 to 100)
Schuit^31^	Flowflex	Netherlands	Testing site	Adult	sympt, HRC, asympt	AN	79.0 (74.7 to 82.8)	97.2 (93.9 to 98.9)
Schuit^31^	MPBio	Netherlands	Testing site	Adult	sympt, HRC, asympt	AN	69.9 (65.1 to 74.4)	98.8 (97.3 to 99.6)
Schuit^31^	Clinitest	Netherlands	Testing site	Adult	sympt, HRC, asympt	AN	70.2 (65.6 to 74.5)	99.3 (97.6 to 99.9)
Schuit^31^	MPBio	Netherlands	Testing site	Adult	sympt, HRC, asympt	OP/nasal	83.0 (78.8 to 86.7)	97.8 (94.3 to 99.4)
Schuit^31^	Clinitest	Netherlands	Testing site	Adult	sympt, HRC, asympt	OP/nasal	77.3 (82.9 to 81.2)	97.0 (93.9 to 98.8)
Schuit^30^	SD Biosensor	Netherlands	Testing site	Adult	sympt, HRC, asympt	NP/OP	68.9 (61.6 to 75.6)	99.5 (99.2 to 99.8)
Schuit^30^	Hangzhou	Netherlands	Testing site	Adult	sympt, HRC, asympt	NP/OP	46.7 (39.3 to 54.2)	99.0 (98.5 to 99.4)
Tonen-Wolyec^32^	Biosynex	France	Testing site	Adult	sympt, HRC, asympt	AN	90.9 (70.8^#^ to 98.9^#^)	100 (95.7^#^ to 100)
Venekamp^33^	FlowFlex	Netherlands	Testing site	Adult	sympt, HRC, asympt	AN	27.5 (21.3 to 34.3)	99.8 (99.3 to 100)
Venekamp^33^	MPBio	Netherlands	Testing site	Adult	sympt, HRC, asympt	AN	20.9 (13.9 to 29.4)	99.8 (99.2 to 100)
Venekamp^33^	Clinitest	Netherlands	Testing site	Adult	sympt, HRC, asympt	AN	25.6 (19.1 to 33.1)	99.9 (99.5 to 100)
Zwart^34^	BD Veritor	Netherlands	Clinical	Adult	sympt., asympt	OP/nasal	61.5 (56.6 to 66.3)	100 (99.8 to 100)
Zwart^34^	BD Veritor	Netherlands	Clinical	Adult	sympt., asympt	AN	50.3 (43.0^#^ to 57.6^#^)	99.7 (99.3 to 99.8)
Zwart^34^	Roche	Netherlands	Clinical	Adult	sympt., asympt	OP/nasal	74.3^#^ (66.6^#^ to 81.1^#^)	99.7 (99.4^#^ to 99.9)

*sympt.* symptomatic, *asympt.* asymptomatic without known contact, *HRC* High risk contact, *AN* Anterior nasal, *OP* Oropharyngeal, *TN* * RT-PCR sample was self-sampled. # Values have been recalculated due to missing or contradictory data.

### Concordance with professional-use Ag-RDTs

**Figure 3:**
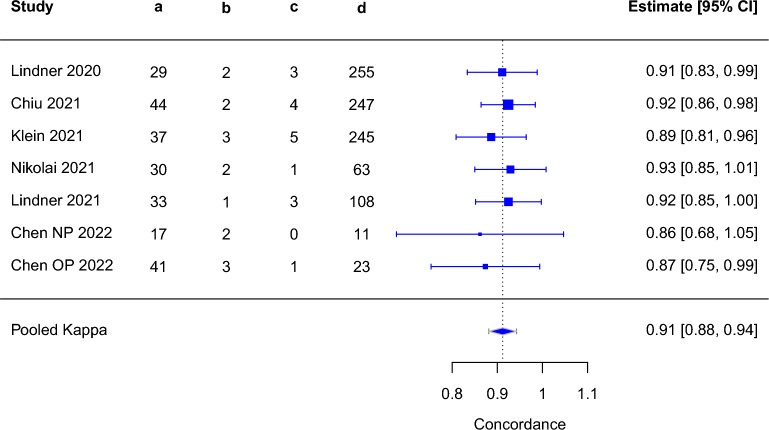
Pooled concordance from self-sampling and self-testing versus professional Ag-RDTs (both sampling and testing performed by professional); Abbreviations: a = self-test & professional test positive; b = self-test positive & professional test negative; c = self-test negative & professional test positive; d = self-test & professional test negative; CI = confidence interval.

The concordance between self-testing and professional testing was only reported in one study, which found high concordance with a kappa of 0.92^11^. The concordance between self-sampling and professional testing was reported in six studies and ranged from 0.86 to 0.93^13,35,39,49,52^. We performed an exploratory analysis of concordance combining datasets from self-sampling and self-testing studies, assuming that sampling is a major driver of differences between self-testing and professional testing. we observed the pooled Cohen’s kappa of 0.91 (95% CI 0.88–0.94) (Fig. 3, Supplementary Table 3).

As only one study was removed^11^, the pooled Cohen’s kappa for self-sampling studies was similar at 0.91 (95% CI 0.88–0.94) (Supplementary Fig. 3, Supplementary Table 3).

### Performance of self-testing and self-sampling in comparison to RT-PCR

When comparing the performance of self-testing using Ag-RDTs to the reference standard, sensitivity ranged widely from 7.7^25^ to 98.2%^28^. Specificity was high, above 99.5% in all datasets.

Across 36 datasets from 31 self-sampling studies, sensitivity again ranged widely from 20.0^43^ to 100%^45^ with wide CIs. Specificity for self-sampling studies ranged from 96.4^39^ to 100%^12^ with narrow CIs. Sensitivity of ≥ 80% was achieved in 15 self-sampling^12,35,37–39,41,45,47–50,52,54,59,60^ and five self-testing studies^11,26,28,31,32^.

A total of 54 datasets assessing 55,115 self-tested or self-sampled samples were eligible for meta-analysis. The meta-analysed summary estimates of sensitivity and specificity across both self-sampling and self-testing datasets were 70.5% (95% CI 64.3–76.0) and 99.4% (95% CI 99.1–99.6), respectively. The pooled sensitivities for self-tested (23 datasets) and self-sampled (31 datasets) samples were 66.1% (95% CI 53.5–76.7) and 73.5% (95% CI 67.4–78.7), respectively.

When only AN sample (40 datasets, 74.1%) were considered, the pooled sensitivity marginally increased to 72.9% (95% CI 65.8–79.0). Test-specific summary estimates of sensitivity were possible for BinaxNow (6 datasets), Standard Q nasal (6 datasets) and Panbio (Abbott, Germany; henceforth called Panbio) (6 datasets), resulting in a sensitivity of 63.5% (95% CI 43.4–79.8), 79.8% (95% CI 66.0–88.9), and 67.7% (95% CI 60.8–73.8), respectively. Data were insufficient for a meta-analysis of other Ag-RDTs or sample types. Supplementary Table S1 provides the full ranges for the clinical performance of each Ag-RDT.

#### IFU-conformity

**Figure 4:**
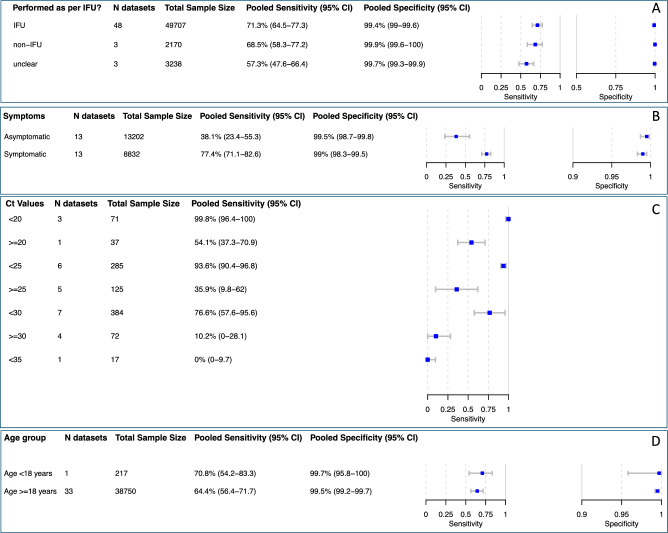
Pooled accuracy of the subgroups (**A**) IFU-conforming sampling, (**B**) symptomatic and asymptomatic persons, (**C**) Ct-value < 25 and < 30, (**D**) age < 18 years and ≥ 18 years. Abbreviations: CI = confidence interval; Ct = cycle threshold; IFU = instructions for use.

Across all self-sampling and self-testing datasets, the overall summary estimate of sensitivity for all IFU-conforming studies was 71.3% (95% CI 64.5–77.3) (Fig. 4A), with marginal differences between self-testing and self-sampling studies (Supplement Figs. 4 and 5). In total three datasets had unclear IFU-conformity with sensitivity ranging from 48.9^24^ to 78.6%^46^.

**Figure 5:**
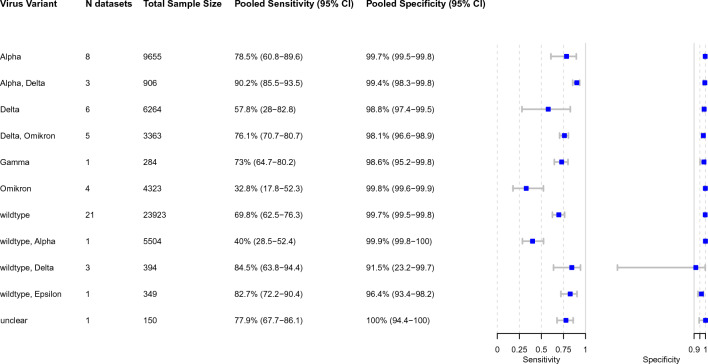
Pooled accuracy for each predominant virus variant. Abbreviations: CI = confidence interval.

In the one study in which participants were observed as they self-tested, the majority of deviation from instructions happened during the sampling procedure, with 41.8% of participants failing to rub the swab against the nasal walls^11^. Another common mistake made during sampling involved too little rotation time in the nose (24.1%)^11^. Squeezing the tube while the swab was still inside and squeezing the tube when the swab was being removed were the steps with most frequent deviations during the testing procedure, at 34.9% and 33.1%, respectively. These deviations, however, did not appear to impact test performance in this study, as performance against RT-PCR (Sensitivity 82.5%) was acceptable and concordance with professional testing was high (kappa 0.91).

#### Presence of symptoms

The summary estimates of sensitivity across all studies were lower in the asymptomatic group compared to the symptomatic group, with 38.1% (95% CI 23.4–55.3) compared to 77.4% (95% CI 71.1–82.6), respectively (Fig. 4B). Specificity was above 99.0% in both subgroups. Self-testing studies, which are included in the pooled analysis, reported a range of sensitivity from 51.0^30^ to 82.5%^11^ in symptomatic persons.

#### Duration of symptoms (DoS)

We were unable to perform a bivariate subgroup meta-analysis for a DoS of more than seven days (DoS > 7) due to an insufficient number of available datasets (n = 1). The reported sensitivity and specificity in this study was 53.8% and 100%, respectively^37^. The pooled estimates of sensitivity and specificity in studies reporting DoS ≤ 7 was 79.4% (95% CI 72.7–84.8) and 99.4% (95% CI 98.9–99.7), respectively.

#### Ct values

For the subgroup analysis based on Ct value range, 22 datasets from nine self-sampling studies were available for univariate meta-analysis. For the Ct value groups < 25 and < 30, the pooled sensitivities were 93.6% (95% CI 90.4–96.8) and 76.6% (95% CI 57.6–95.6), respectively (Fig. 4C).

Testing using self-sampling in patients who had samples with Ct values ≥ 25 and ≥ 30 showed a broader range, with pooled sensitivities of 35.9% (95% CI 9.8–62.0) and 10.2% (0.0–28.1), respectively.

One self-testing study reported a sensitivity of 85.0% and a specificity of 99.1% when only samples with high viral load (≥ 7.0 log_10_ SARS-CoV-2 RNA copies/mL) were analyzed^11^.

#### Age

Across all the studies included in the review, we had 32 datasets with samples from people aged 18 years and older (‘ ≥ 18 years’), achieving a pooled sensitivity of 65.5% (95% CI 57.8–72.4) (Fig. 4D). For the ‘ < 18 years’ group, a meta-analysis was not possible, as only three datasets were available for this age group. However, the reported sensitivity in these three datasets had a comparable range to that in the ‘ ≥ 18 years’ group (71.4^48^ to 92.3%^47^). The pooled specificity was 99.6% (95% CI 99.2–99.8) in the ‘ ≥ 18 years’ group and was above 99.6% in all datasets in the ‘ < 18 years’ group.

#### Virus variant

VoC could be determined for 53 datasets out of 54, wild type observed in 21 datasets (39.6% of all datasets). The pooled sensitivity across these 21 datasets was 69.8% (95% CI 62.5–76.3) and the pooled specificity was 99.7% (95% CI 99.5–99.8). The highest sensitivity was found across studies conducted when the alpha VoC (8 datasets, 15.1%) was predominant, with 78.5% (95% CI 60.8–89.6). Across studies conducted during an Omicron wave (4 datasets, 7.5%), the pooled sensitivity was significantly lower with 32.8% (95% CI 17.8–52.3). When Delta (6 datasets, 11.3%) was predominant, the pooled sensitivity increased to 57.8% (95% CI 28.0–82.8). However, in other studies when Delta and Omicron were predominant had a pooled sensitivity of 76.1% (95% CI 70.7–80.7) (Fig. 5).

Self-testing studies showed similar pooled estimates for sensitivity for wild type, combined Delta/Omicron, and alpha VoC with 62.6% (95% CI 52.2–72.0), 76.1% (95% CI 70.7–80.7), and 85.3% (54.0–96.6), respectively.

#### Middle-Income Countries (MIC) vs. High-Income Countries (HIC)

Studies conducted in HIC accounted for 44 datasets (53,090 samples), resulting in a pooled sensitivity and specificity of 67.6% (95% CI 60.5–74.0) and 99.5% (95% CI 99.3–99.7), respectively. In contrast, studies from MIC (10 datasets; 2025 samples) had higher sensitivity and comparable specificity with 81.0% (95% CI 70.4–88.4) and 98.1% (95% CI 93.9–99.4), respectively (Supplement Figs. 6 and 7).

#### Sensitivity analysis

When excluding case–control studies (5 datasets), the sensitivity remained comparable to the overall pooled sensitivity estimate with 69.5% (95% CI 62.8–75.5) (Supplement Fig. 8).

Datasets from manufacturer-independent studies (40 datasets; 20 self-testing studies) achieved an accuracy comparable to the overall summary estimates with a pooled sensitivity of 66.5% (95% CI 59.2–73.1) and a pooled specificity of 99.5% (95% CI 99.1–99.7) (Supplement Fig. 9). Excluding preprints (5 datasets) resulted in no substantial change in sensitivity (69.9% [95% CI 63.2–75.8]) and specificity (99.4% [95% CI 99.0–99.6]) (Supplement Fig. 10).

#### Certainty of evidence (CoE)

We found CoE to be high for specificity and sensitivity, and low for concordance and user errors. As for ‘imprecision’, we downgraded the CoE for concordance by one point due to the low number of studies and small sample size. For studies assessing concordance and user errors, ‘inconsistency’ was rated ‘serious’ and consequently also downgraded by one point, since there was only one study available (Table 3).

**Table 3:** GRADE table: Should COVID-19 self-testing, defined as self-sampling, processing of the sample and self-readout using Ag-RDTs, be offered as an additional approach to professionally administered testing services? The following table summarizes the certainty of evidence according to the GRADE approach.

Certainty assessment							Impact	Certainty	Importance
№ of studies	Study design	Risk of bias	Inconsistency	Indirectness	Imprecision	Other considerations			
*Accuracy—sensitivity (Ag-RDT self-testing vs. rRT-PCR)*									
23^11,24–34^	Observational studies	Not serious^a^	Not serious^b^	Not serious^c^	Not serious^d^	None	Normalized to a study population with 1000 participants and 10% prevalence, 66 true positive and 34 false negative self-testing results were reported. Pooled sensitivity was 66.1% (95% CI 53.5 to 76.7)	⨁⨁⨁⨁ High	CRITICAL
*Accuracy—specificity (Ag-RDT self-testing vs. rRT-PCR)*									
23^11,24–34^	Observational studies	Not serious^a^	Not serious^b^	Not serious^c^	Not serious^d^	None	Normalized to a study population with 1000 participants and 10% prevalence, 874 true negative and 2 false positive self-testing results were reported. Pooled specificity was high with 99.5% (95% CI 99.1 to 99.7)	⨁⨁⨁⨁ High	CRITICAL
*Accuracy—concordance (Ag-RDT self-testing vs. Ag-RDT performed by professionals)*									
1^11^	Observational studies	Not serious^a^	Serious^b^	Not serious^c^	Serious^d^	None	Kappa: 0.92 (out of 1.00); (95% CI 0.89 to 0.95)	⨁⨁◯◯ Low	CRITICAL
*Accuracy—Proportion of user errors*									
1^11^	Observational studies	Not serious^a^	Serious^b^	Not serious^c^	Not serious^e^	None	15.5% of the sampling steps and 15.0% of testing steps, were found to have deviations by study participants. However, these did not impede the self-test's performance	⨁⨁◯◯ Low	IMPORTANT

Explanation: ^a^We used QUADAS-2 to assess risk of bias. The studies enrolled patients consecutively and assessed the self-testing, defined as self-sampling and self-performing the Ag-RDT, results blinded to the reference standard result (rRT-PCR or prof. Ag-RDT testing). While for one study it was not clear whether all self-tests were performed as per manufacturer’s instructions, this was ensured in the other. Furthermore, we could not detect any potential bias resulting from the study flow and timing. Therefore, we did not downgrade the quality of evidence for this criterion.

^b^The heterogeneity/inconsistency in findings, as shown by the wide-ranging point estimates with only marginally overlapping confidence intervals, is likely to originate from differences in the study population. This is strengthened by the fact that the head-to-head comparison between self-testing and professionally testing on the same study population shows similar performance of Ag-RDTs. However, as there are only a few studies available for concordance and one study for user errors, we downgrade for these two outcomes by one.

^c^Following current guidance from the GRADE guideline, we do not downgrade by one point for all studies but acknowledge that the study populations are not fully representative of the populations of interest. Furthermore, the intervention did not differ from the one of interest and outcomes were reported directly, therefore indirectness was judged 'not serious'.

^d^The number of studies and sample size were small, and only one study reported on concordance between self-testing and professionally testing using Ag-RDTs.

^e^For this outcome only qualitative data, or quantitative data in isolated studies in well-described but not comparable settings were available, therefore the criterion 'imprecision' is negligible and rated as 'not serious'.

## Discussion

Our systematic review and meta-analysis found that concordance between self-testing/self-sampling and professional testing using Ag-RDTs is very high with a pooled Cohen’s kappa of 0.91 (95% CI 0.88–0.94). Compared to RT-PCR, sensitivity of self-testing/self-sampling across all studies included in our review compared to RT-PCR (70.5% [95% CI 64.3–76.0]) was estimated to be almost the same as that of Ag-RDTs when performed by professionals (72.0%^8^). The summary point estimate of sensitivity for self-testing studies (66.1% [95% CI 53.5–76.7]) was also comparable to that of professional-conducted Ag-RDT with overlapping CIs.

Pooled sensitivity across self-testing and self-sampling studies increased to 77.4% (95% CI 71.1–82.6) in symptomatic persons, which is in line with the results of earlier reports that showed that presence of symptoms was a key variable affecting sensitivity of Ag-RDT and correlated with viral load^8,65^. Thus, neither overall nor symptomatic pooled sensitivity achieved WHO sensitivity targets of ≥ 80%^10^. Notably, a recent meta-analysis found a pooled sensitivity of 91.1% for Ag-RDTs with self-collected nasal samples^66^.

The results of subgroup analysis based on Ct values are consistent with those of earlier studies, suggesting that viral load is the main determinant of test sensitivity, irrespective of the sampling procedure or the person administering the test^8^. Because Ag-RDTs detect the vast majority of SARS-CoV-2-infected persons with high viral load, self-testing becomes a valuable public health tool for identifying individuals who might be at risk of spreading the virus, especially when RT-PCR testing is not accessible. This approach aids in creating safer environments for reopening schools, workplaces, and organizing large gatherings amid the pandemic.

In addition, it is worth noting that in most cases (60.0% of datasets), the sampling process was unsupervised, which implies the general applicability of our findings to unobserved home-testing. Moreover, even though deviations from the IFU did occur in some cases, this did not appear to have an impact on test performance^11^.

Although limited, the data on deviations from sampling and testing procedures demonstrated that most instruction deviations occurred during sampling, supporting our approach to conduct a pooled exploratory analysis of self-sampling and self-testing. This was additionally bolstered by a positive self-judgement of test execution and interpretation, showing confidence of lay-users to perform Ag-RDTs reliably^24^. Moreover, one study reported that healthcare professionals and laypersons had a high level of readout agreement when clear instructions with illustrations were available^11^. It is, however, crucial to note that the observed sampling deviations are more likely to affect test sensitivity than specificity, because poor sampling is likely to result in decreased sample quality, and thus lower viral load, leading to false negative results. Nevertheless, the results of the sensitivity analysis showed that the pooled sensitivity estimate for self-testing studies is still lower than that for self-sampling studies, which suggests that self-sampling is not the only variable influencing the differences between self-testing and professional testing. To fully understand all the variables and how they affect test performance, more research is necessary.

Our subgroup analysis on VoC showed higher sensitivity when Delta and Omicron (76.1% [95% CI 70.7–80.7]) were predominant compared to Omicron (32.8% [95% CI 17.8–52.3%]) alone. However, the four data sets for Omicron analysis emerged from two studies^33,63^. Both studies included primarily asymptomatic persons and had a > 92% vaccination rate, resulting likely in a lower viral load and thus affecting test sensitivity ^33,63^.

Our study has several strengths. We thoroughly assessed the included studies with the QUADAS-2 tool using an a-priori developed interpretation guide. In addition, our review was supported by an independent methodologist and followed rigorous methods, aligning with other WHO-commissioned reviews for self-testing. Furthermore, we report on both peer-reviewed articles and preprints from a period that nearly covers the whole pandemic. Another strength of this study lies within our subgroup analyses that provide a clearer picture of the accuracy of self-sampling and self-testing across different populations and testing approaches.

Our systematic review is, however, limited by the small number of studies that were deemed eligible (particularly those evaluating self-testing) as well as the shortcomings of these studies as revealed by the quality assessment. The degree to which study participants with a relatively high rate of symptomatic individuals with prior training or testing experience are representative of the general population is another drawback. Furthermore, the majority of studies were conducted in HIC; at the same time, populations in MIC, particularly those with a high-burden of HIV, were likely to have more experience with self-testing compared to HIC at the beginning of the pandemic ^3^. Recent reports find good concordance between COVID-19 self-testing and professionally-conducted Ag-RDTs in a middle-income country ^67^. Although there are differences that cannot be accounted for in this meta-analysis, our exploratory analysis found a higher pooled estimate of sensitivity in MIC compared to HIC.

## Conclusion

Self-testing and/or self-sampled testing using Ag-RDTs likely achieves similar accuracy as professional-use Ag-RDTs. In the light of the evidence presented in this review and other supporting studies, the WHO recommends COVID-19 self-testing to scale-up testing capacity ^68,69^. Further evidence is required to assess the impact of testing strategies including self-testing on the population-level control of SARS-CoV-2 transmission.

### Supplementary Information

Supplementary Information 1.

Supplementary Table 1.

Supplementary Table 2.

Supplementary Table 3.

## Data Availability

The raw data is available under https://doi.org/10.11588/data/P9JEPG.

## Abbreviations

Ag-RDT: Antigen detection rapid diagnostic test
AN: Anterior nasal
CI: Confidence interval
CoE: Certainty of evidence
Ct: Cycle threshold
DOS: Duration of symptoms
FN: False negative
FP: False positive
HIC: High-income countries
IFU: Instructions for use
MIC: Middle-income countries
NMT: Nasal mid-turbinate
NP: Nasopharyngeal
NPA: Negative percentage agreement
OP: Oropharyngeal
OPA: Overall percentage agreement
POC: Point of care
PPA: Positive percentage agreement
PRISMA: Preferred Items for Systematic Reviews and Meta-analysis
RCT: Randomized controlled trial
RT-PCR: Reverse transcription polymerase chain reaction
TN: True negative
TP: True positive
VoC: Variant of concern
WHO: World Health Organization
