# Supplementary material for: Comparing SARS-CoV-2 antigen-detection rapid diagnostic tests for COVID-19 self-testing/self-sampling with molecular and professional-use tests: a systematic review and meta-analysis
Source: Sci Rep. 2023 Dec 11;13:21913. doi: 10.1038/s41598-023-48892-x (PMC10713601; doi:10.1038/s41598-023-48892-x)
Supplement: Supplementary file 1 — Supplementary Information 1. [file 41598_2023_48892_MOESM1_ESM.docx]

**Supplementary Material to**

**Performance of SARS-CoV-2 antigen-detection rapid diagnostic tests for COVID-19 self-testing and self-sampling in comparison to molecular and professional-use antigen tests: A systematic review and meta-analysis**

Stephan Katzenschlager^1^, Lukas E. Brümmer^2^, Stephani Schmitz^2,3^, Hannah Tolle^2^, Katharina Manten^1,2^, Mary Gaeddert^2^, Christian Erdmann^4^, Andreas Lindner^5^, Frank Tobian^2^, Maurizio Grilli^6^, Nira R. Pollock^7^, Aurélien Macé^8^, Berra Erkosar^8^, Sergio Carmona^8^, Stefano Ongarello^8^, Cheryl C. Johnson^9^, Jilian A. Sacks^10^, Claudia M. Denkinger^2, 11^, Seda Yerlikaya^2^ *

* Corresponding author

[seda.yerlikaya@uni-heidelberg.de](mailto:seda.yerlikaya@uni-heidelberg.de)

Division of Infectious Disease and Tropical Medicine, Center for Infectious Diseases, Heidelberg University Hospital

Im Neuenheimer Feld 324

69120 Heidelberg, Germany

1) Department of Anesthesiology, Heidelberg University Hospital, Heidelberg, Germany

2) Division of Infectious Disease and Tropical Medicine, Center for Infectious Diseases, Heidelberg University Hospital, Heidelberg, Germany

3) Department of Developmental Biology, Erasmus Medical Center, Rotterdam, Netherlands

4) FH Muenster University of Applied Sciences, Muenster, Germany

5) Charité – Universitätsmedizin Berlin, corporate member of Freie Universität Berlin, Humboldt- Universität zu Berlin, and Berlin Institute of Health; Institute of Tropical Medicine and International Health, Berlin, Germany

6) Library, University Medical Center Mannheim, Mannheim, Germany

7) Department of Laboratory Medicine, Boston Children’s Hospital, Boston, Massachusetts, United States of America

8) FIND, Geneva, Switzerland

9) Global HIV, Hepatitis and STIs Programmes, World Health Organization, Geneva, Switzerland

10) Department of Epidemic and Pandemic Preparedness and Prevention, World Health Organization, Geneva, Switzerland

11) German Center for Infection Research (DZIF), partner site Heidelberg University Hospital, Heidelberg, Germany

Table of content

[Supplement PRISMA Checklist 4](#_Toc152845897)

[Supplement Text 1 – Study Protocol 10](#_Toc152845898)

[Supplement Text 2 – Search Strategy 20](#_Toc152845899)

[Review questions 20](#_Toc152845900)

[Definitions 20](#_Toc152845901)

[Strategie 20](#_Toc152845902)

[Searched Databases 20](#_Toc152845903)

[PubMed 20](#_Toc152845904)

[Web of Science Core Collection 22](#_Toc152845905)

[Bio_MedRxiv 23](#_Toc152845906)

[Supplement Text 3 – QUADAS assessment interpretation guide 24](#_Toc152845907)

[Supplement Figure 1 – QUADAS 28](#_Toc152845908)

[Supplement Figure 2 – Funnel Plot 28](#_Toc152845909)

[Supplement Figure 3 – Pooled Cohen’s kappa 29](#_Toc152845910)

[Supplement Figure 4 – Accuracy for self-testing studies according to IFU-conformity 31](#_Toc152845911)

[Supplement Figure 5 – Accuracy for self-sampled studies according to IFU-conformity 31](#_Toc152845912)

[Supplement Figure 6 – Accuracy for high income countries 31](#_Toc152845913)

[Supplement Figure 7 – Accuracy for middle income countries 32](#_Toc152845914)

[Supplement Figure 8 – Sensitivity analysis excluding case-control studies 32](#_Toc152845915)

[Supplement Figure 9 – Sensitivity analysis excluding manufacturer-dependent studies 32](#_Toc152845916)

[Supplement Figure 10 – Sensitivity analysis excluding preprints 33](#_Toc152845917)

# Supplement PRISMA Checklist

| **Section and Topic** | **Item #** | **Checklist item** | **Location where item is reported** |
| --- | --- | --- | --- |
| **TITLE** | | |  |
| Title | 1 | Identify the report as a systematic review. | Title of paper |
| **ABSTRACT** | | |  |
| Abstract | 2 | See the PRISMA 2020 for Abstracts checklist. | Abstract Checklist |
| **INTRODUCTION** | | |  |
| Rationale | 3 | Describe the rationale for the review in the context of existing knowledge. | Introduction, Paragraph 2 |
| Objectives | 4 | Provide an explicit statement of the objective(s) or question(s) the review addresses. | Introduction, Paragraph 2 |
| **METHODS** | | |  |
| Eligibility criteria | 5 | Specify the inclusion and exclusion criteria for the review and how studies were grouped for the syntheses. | Subsection “Eligibility criteria” |
| Information sources | 6 | Specify all databases, registers, websites, organisations, reference lists and other sources searched or consulted to identify studies. Specify the date when each source was last searched or consulted. | Subsection “Search strategy” |
| Search strategy | 7 | Present the full search strategies for all databases, registers and websites, including any filters and limits used. | Supplementary File “S2 Text Search Strategy” |
| Selection process | 8 | Specify the methods used to decide whether a study met the inclusion criteria of the review, including how many reviewers screened each record and each report retrieved, whether they worked independently, and if applicable, details of automation tools used in the process. | Selection process was done accordingly to our previous analysis. First Paragraph in Methods section. |
| Data collection process | 9 | Specify the methods used to collect data from reports, including how many reviewers collected data from each report, whether they worked independently, any processes for obtaining or confirming data from study investigators, and if applicable, details of automation tools used in the process. | Data collection process was done accordingly to our previous analysis. First Paragraph in Methods section. |
| Data items | 10a | List and define all outcomes for which data were sought. Specify whether all results that were compatible with each outcome domain in each study were sought (e.g. for all measures, time points, analyses), and if not, the methods used to decide which results to collect. | Supplementary File “S2 Table Parameters” |
|  | 10b | List and define all other variables for which data were sought (e.g. participant and intervention characteristics, funding sources). Describe any assumptions made about any missing or unclear information. | Supplementary Files S1 Table and https://doi.org/10.11588/data/P9JEPG |
| Study risk of bias assessment | 11 | Specify the methods used to assess risk of bias in the included studies, including details of the tool(s) used, how many reviewers assessed each study and whether they worked independently, and if applicable, details of automation tools used in the process. | Study risk of bias assessment was done accordingly to our previous analysis. First Paragraph in Methods section.  Subsection “Assessment of methodological quality” |
| Effect measures | 12 | Specify for each outcome the effect measure(s) (e.g. risk ratio, mean difference) used in the synthesis or presentation of results. | Subsection “Statistical analysis and data synthesis” Paragraph 2 |
| Synthesis methods | 13a | Describe the processes used to decide which studies were eligible for each synthesis (e.g. tabulating the study intervention characteristics and comparing against the planned groups for each synthesis (item #5)). | Subsection “Statistical analysis and data synthesis” Paragraph 2 |
|  | 13b | Describe any methods required to prepare the data for presentation or synthesis, such as handling of missing summary statistics, or data conversions. | Subsection “Statistical analysis and data synthesis” Paragraph 1 |
|  | 13c | Describe any methods used to tabulate or visually display results of individual studies and syntheses. | Subsection “Statistical analysis and data synthesis” Paragraph 3 |
|  | 13d | Describe any methods used to synthesize results and provide a rationale for the choice(s). If meta-analysis was performed, describe the model(s), method(s) to identify the presence and extent of statistical heterogeneity, and software package(s) used. | Subsection “Statistical analysis and data synthesis” Paragraph 2 and 3 |
|  | 13e | Describe any methods used to explore possible causes of heterogeneity among study results (e.g. subgroup analysis, meta-regression). | Subsection “Statistical analysis and data synthesis” Paragraph 3 and 4 |
|  | 13f | Describe any sensitivity analyses conducted to assess robustness of the synthesized results. | Subsection “Sensitivity analysis” |
| Reporting bias assessment | 14 | Describe any methods used to assess risk of bias due to missing results in a synthesis (arising from reporting biases). | Subsection “Statistical analysis and data synthesis” Paragraph 5 |
| Certainty assessment | 15 | Describe any methods used to assess certainty (or confidence) in the body of evidence for an outcome. | Subsection “Assessment of certainty of evidence (CoE)” |
| **RESULTS** | | |  |
| Study selection | 16a | Describe the results of the search and selection process, from the number of records identified in the search to the number of studies included in the review, ideally using a flow diagram. | Fig1 PRISMA Flow Diagram |
|  | 16b | Cite studies that might appear to meet the inclusion criteria, but which were excluded, and explain why they were excluded. | Section Results, Paragraph 1 and Figure 1 |
| Study characteristics | 17 | Cite each included study and present its characteristics. | Supplementary Files “S1 Table” and https://doi.org/10.11588/data/P9JEPG |
| Risk of bias in studies | 18 | Present assessments of risk of bias for each included study. | Supplementary File “S1 Figure QUADAS” and https://doi.org/10.11588/data/P9JEPG |
| Results of individual studies | 19 | For all outcomes, present, for each study: (a) summary statistics for each group (where appropriate) and (b) an effect estimate and its precision (e.g. confidence/credible interval), ideally using structured tables or plots. | Subsection “Study description” and Table 1 and Supplementary File “S1 Table” |
| Results of syntheses | 20a | For each synthesis, briefly summarise the characteristics and risk of bias among contributing studies. | Subsection “Methodological quality of all included studies” and Figure 2AB |
|  | 20b | Present results of all statistical syntheses conducted. If meta-analysis was done, present for each the summary estimate and its precision (e.g. confidence/credible interval) and measures of statistical heterogeneity. If comparing groups, describe the direction of the effect. | Subsection “Concordance with professional-use Ag-RDTs” and Figure 3  Subsection “Performance of self-testing and self-sampling in comparison to RT-PCR” and Figure 4A-D |
|  | 20c | Present results of all investigations of possible causes of heterogeneity among study results. | Subsections “IFU conformity”, “Presence of symptoms”, “Duration of Symptoms”, “Ct Values”, “Age”, “VoC”, and “LMIC vs. HIC” |
|  | 20d | Present results of all sensitivity analyses conducted to assess the robustness of the synthesized results. | Subsection “Sensitivity Analysis” |
| Reporting biases | 21 | Present assessments of risk of bias due to missing results (arising from reporting biases) for each synthesis assessed. | Supplementary Files “S1 Table” and https://doi.org/10.11588/data/P9JEPG |
| Certainty of evidence | 22 | Present assessments of certainty (or confidence) in the body of evidence for each outcome assessed. | Subsection “Certainty of Evidence (CoE)” |
| **DISCUSSION** | | |  |
| Discussion | 23a | Provide a general interpretation of the results in the context of other evidence. | Paragraph 1,2,3 |
|  | 23b | Discuss any limitations of the evidence included in the review. | Paragraph 5 |
|  | 23c | Discuss any limitations of the review processes used. | Paragraph 5 |
|  | 23d | Discuss implications of the results for practice, policy, and future research. | Section “Conclusion” |
| **OTHER INFORMATION** | | |  |
| Registration and protocol | 24a | Provide registration information for the review, including register name and registration number, or state that the review was not registered. | Abstract, Section “Methods” First Paragraph |
|  | 24b | Indicate where the review protocol can be accessed, or state that a protocol was not prepared. | Abstract, Section “Methods” and Supplementary Files “S1 Text Study Protocol” |
|  | 24c | Describe and explain any amendments to information provided at registration or in the protocol. | N/A |
| Support | 25 | Describe sources of financial or non-financial support for the review, and the role of the funders or sponsors in the review. | Section “Funding” |
| Competing interests | 26 | Declare any competing interests of review authors. | N/A |
| Availability of data, code and other materials | 27 | Report which of the following are publicly available and where they can be found: template data collection forms; data extracted from included studies; data used for all analyses; analytic code; any other materials used in the review. | Data is available under: https://doi.org/10.11588/data/P9JEPG |

*From:*  Page MJ, McKenzie JE, Bossuyt PM, Boutron I, Hoffmann TC, Mulrow CD, et al. The PRISMA 2020 statement: an updated guideline for reporting systematic reviews. BMJ 2021;372:n71. doi: 10.1136/bmj.n71

For more information, visit: <http://www.prisma-statement.org/>

**Prisma 2020 for Abstracts Checklist**

| **Section and Topic** | **Item #** | **Checklist item** | **Reported (Yes/No)** |
| --- | --- | --- | --- |
| **TITLE** | | |  |
| Title | 1 | Identify the report as a systematic review. | Yes |
| **BACKGROUND** | | |  |
| Objectives | 2 | Provide an explicit statement of the main objective(s) or question(s) the review addresses. | Yes |
| **METHODS** | | |  |
| Eligibility criteria | 3 | Specify the inclusion and exclusion criteria for the review. | No |
| Information sources | 4 | Specify the information sources (e.g. databases, registers) used to identify studies and the date when each was last searched. | Yes |
| Risk of bias | 5 | Specify the methods used to assess risk of bias in the included studies. | Yes |
| Synthesis of results | 6 | Specify the methods used to present and synthesise results. | Yes |
| **RESULTS** | | |  |
| Included studies | 7 | Give the total number of included studies and participants and summarise relevant characteristics of studies. | Yes |
| Synthesis of results | 8 | Present results for main outcomes, preferably indicating the number of included studies and participants for each. If meta-analysis was done, report the summary estimate and confidence/credible interval. If comparing groups, indicate the direction of the effect (i.e. which group is favoured). | Yes |
| **DISCUSSION** | | |  |
| Limitations of evidence | 9 | Provide a brief summary of the limitations of the evidence included in the review (e.g. study risk of bias, inconsistency and imprecision). | Yes |
| Interpretation | 10 | Provide a general interpretation of the results and important implications. | Yes |
| **OTHER** | | |  |
| Funding | 11 | Specify the primary source of funding for the review. | No |
| Registration | 12 | Provide the register name and registration number. | Yes |

*From:*  Page MJ, McKenzie JE, Bossuyt PM, Boutron I, Hoffmann TC, Mulrow CD, et al. The PRISMA 2020 statement: an updated guideline for reporting systematic reviews. BMJ 2021;372:n71. doi: 10.1136/bmj.n71

For more information, visit: <http://www.prisma-statement.org/>

# Supplement Text 1 – Study Protocol

1. TITLE

Feasibility and accuracy of self-tested antigen rapid diagnostic tests against SARS-CoV-2: a systematic review and meta-analysis

2. BACKGROUND AND RATIONALE

Point of care (POC) antigen tests have been proven to be a valuable tool in the fight against COVID-19^1^. Such tests are widely used for screening as well as monitoring and diagnostic purposes. Within up to half an hour, depending on the test used, the operator receives the test result. In comparison, results from reverse transcription polymerase chain reaction (RT-PCR) can take up to 48 hours or longer. RT-PCR remains the gold standard in diagnosing SARS-CoV-2.

Performance of antigen rapid diagnostic tests (Ag-RDT) varies between different manufacturers, sampling types and viral load present. In recent meta-analyses overall sensitivity was highest in patients with a Ct value ≤25 and in patients within the first week of symptoms^2,3^.

Ag-RDT are easy to perform and studies have suggested that they can be conducted by lay operators. Errors in sampling technique are common, but overall agreement has shown promising results when both swabs were assessed comparing RT-PCR^4–6^.

Self-sampling and self-testing would reduce the need for protective personal equipment and the risk of infection for healthcare workers^5^. The utility of these tests for pandemic control through mass testing or testing to protect (e.g. in high-risk settings such as hospitals), to release (e.g. contact testing) and to enable (e.g. regular school or workplace testing) has been suggested in different studies^7–10^.

With the proposed systematic review and meta-analysis, we will give an overview on the currently published accuracy evaluations on self-sampled and self-tested SARS-CoV-2 antigen POC diagnostics against the PCR reference standard. Furthermore, we assess concordance where self-testing or self-sampling was compared to professionally sampled or tested participants using Cohen’s kappa, positive predictive value, negative predictive value for head to head studies. Where possible, we will perform a meta-analysis, summarizing the sensitivity and specificity and concordance of tests that have been evaluated by multiple studies and analyze aspects likely to affect the Ag-RDTs accuracy (e.g. viral load).

The quality of the studies will be evaluated. Information about self-sampling and self-testing process and its ease of use/feasibility will be assessed and described in a narrative.

For the purpose of the WHO Guideline Development Meeting, we will focus on self-testing.

3. REVIEW QUESTION

To (1) assess the feasibility and accuracy of commercially available Ag-RDTs with self-sampled and self-tested samples compared to RT-PCR and (2) assess the concordance between self-sampled and self-tested samples compared to professional samples and (3) ease-of-use and feasibility of self-sampling and self-testing.

4. CRITERIA FOR CONSIDERING STUDIES FOR THE REVIEW

- 1. **Types of studies (designs):**

Clinical studies evaluating the accuracy of self-sampled or self-tested antigen POC tests for SARS CoV 2 detection against a RT-PCR as reference standard. Clinical studies evaluating the concordance between self-sampled or self-tested samples compared to professional tested samples.

We will consider retrospective or prospective cohort or nested cohort studies, or cross-sectional studies, as well as randomized studies. Publications with less than 10 samples will be excluded. To present the latest available data, we will consider both peer reviewed publications and preprints as eligible.

- 1. **Types of participants:**

Patients of all age-groups suspected of having or confirmed with SARS-CoV-2. All countries. We will exclude any studies in which patients are being tested for monitoring or end of quarantine. Although the size threshold of 10 is arbitrary, such small studies are likely to give unreliable estimates of sensitivity or specificity.

- 1. **Index test:**

Antigen POC diagnostic for SARS-CoV-2 of any type

- 1. **Target conditions**

Persons presumed to have COVID-19, screening independent of symptoms, high risk contacts

- 1. **Reference Standard:**

For the purposes of this review, we considered RT-PCR to be the ‘reference standard’ against which the rapid tests are compared. RT-PCR is the most direct reference standard for an antigen test as RNA to be highly correlated with Antigen quantities^11^. The result of further RT-PCR analysis of discrepant cells (samples with results disagreeing on the rapid test and the RT-PCR) will also considered in sensitivity analyses. We will consider results from retesting with a second test in a sensitivity analysis.

In studies reporting on comparison between self- and professional tested samples in the absence of a RT-PCR, we will report concordance to professional samples.

5. Criteria for considering studies for the METANANLYSIS:

We will only consider all studies that have been included into the systematic review as eligible for the meta-analysis. We will provide meta-analysis for clinical accuracy. In addition, we will only provide a test specific meta-analysis for index tests that have been evaluated by at least 4 studies.

6. SEARCH METHODS

We will perform an electronic search in the databases PubMed and Web of Science using an automated search algorithm. The main search terms are “Severe Acute Respiratory Syndrome Coronavirus 2”, “COVID-19”, “Betacoronavirus”, “Coronavirus” and “Point of Care Testing”. A librarian defined the search. We will search for any literature published between December 01^st^ 2019 and November 16^th^ 2021. No language restrictions will be applied.

7. REVIEW METHODS

- 1. **Study selection methods:**

Two independent authors (LEB and CE or LEB and SS or SS and CE or SK and HT) will review the titles and abstracts of all publications returned by the search algorithm (screen 1). Any publication selected for inclusion by one of the authors will be considered as potentially eligible. All potentially eligible publications will be reviewed again in detail by the same authors (screen 2). After the detailed review, both authors will independently decide which publications to include into the systematic review. Any disputes will be solved by discussion or by a third author (CMD).

- 1. **Data extraction methods:**

We extracted only data for Ag-RDT results at the time of diagnosis. If multiple different RT-PCR tests were performed on one sample within one study, we utilized the data from the best-performing assay.

If a study contributed data to more than one analysis (e.g., two different alternative sample types in one study used for the Ag-RDT), it was considered as two or more datasets. In addition to data on test performance, we extracted data for factors likely to affect test performance: (1) asymptomatic versus symptomatic (2) symptom duration prior to testing (3) Type of RT-PCR (4) populations (e.g. pediatric versus adults); (5) site of sampling; (6) viral load; (7) Ct-value; (8) Sampling (e.g. lay versus professional; if lay person sampling: supervised, non-supervised); (9) Test conducted (e.g. self or professional); (10) use case (e.g. universal screening, targeted screening, diagnostic use case); (11) Setting (e.g. hospitals, schools, work environment); (12) testing procedure (e.g. self-sampling or self-testing)

Where possible, we separately extracted data according to viral load, and for molecular assays, before and after re-analysis of samples in discrepant cells.

- 1. **Quality assessment methods [risk of bias in individual studies] and how quality data will be used:**

We will assess the quality of each study by applying the QUADAS-2 tool^12^. The tool consists out of four domains: patient selection, index test, reference standard, and flow and timing. For each domain, the risk of bias is analyzed using different signaling questions. Beyond the risk of bias, the tool also evaluates the applicability of the study design to the research question for every domain. We have adapted the tool for the present review as follows:

**Domain 1 Patient Selection:**

Risk of Bias: Could the selection of patients have introduced bias?

• Signaling question 1: Was a consecutive or random sample of patients or specimens enrolled?

Score ‘yes’ if the study enrolled a consecutive or random sample of eligible patients; ‘no’ if the study selected patients by convenience, and ‘unclear’ if the study did not report the manner of patient selection or unable to tell.

• Signaling question 2: Was a case-control design avoided?

We scored ’yes’ to all included studies given that we are excluding case-control study designs.

• Signaling question 3: Did the study avoid inappropriate exclusions?

We scored ’yes’ to studies which included: all participants a) regardless of symptoms or b) duration of symptoms and c) prior testing (e.g. CXR). We scored ’no’ if studies excluded participants on the basis of symptoms or duration of symptoms or prior testing. We scored ’unclear’ if we could not tell.

- Risk of Bias is scored ‘low concern’ if studies score ‘yes’ on all the question, ‘unclear concern’ if questions are answered with ‘yes’ and ‘unclear’, ‘intermediate concern’ if one question is answered with ‘no’, ‘high concern’ if two or more questions are answered with ‘no’.

Applicability: Are there concerns that the included patients and setting do not match the review question? We were interested in how Ag-RDT performs in patients whose specimens were evaluated as they would be in routine practice. We expected to score most studies as ’low concern’ since we planned to determine test accuracy only for COVID diagnosis. We scored ‘high concern’ if Ag-RDT were evaluated for end of quarantine evaluation or monitoring.

**Domain 2: Index Test**

Risk of Bias: Could the conduct or interpretation of the index test have introduced bias?

• Signaling question 1: Were the index test results interpreted with knowledge of the results of the reference standard?

We answered ’yes’ if the study interpreted the result of Ag-RDT blinded to the result of the reference standard; we answered ’no’ if the study did not interpret the result of Ag-RDT blinded to the result of the reference standard. We answered ’yes’ for studies in which Ag-RDT was performed on fresh specimens, since reference standard results would be unavailable at the time of test interpretation. We answered ’unclear’ if stored specimens were tested or we could not tell if the index test results were interpreted without knowledge of the reference standard results.

• Signaling question 2: If a threshold was used, was it prespecified?

We answered ’yes’ if the threshold was prespecified or if the tests was performed by IFU. We scored ’no’ if the threshold was not prespecified, and ’unclear’ if we could not determine if the threshold was prespecified or not.

- Risk of Bias is scored ‘low concern’ if studies score ‘yes’ on all the question, ‘unclear concern’ if questions are answered with ‘yes’ and ‘unclear’, ‘intermediate concern’ if one question is answered with ‘no’, ‘high concern’ if two or more questions are answered with ‘no’.

Applicability: Are there concerns that the index test, its conduct, or its interpretation differ from the review question? If index test methods vary from those specified in the review question, concerns about applicability may exist. We judged ’high concern’ if the test procedure was inconsistent with the manufacturer recommendations, ’low concern’ if the test procedure was consistent with the manufacturer recommendations, and ’unclear concern’ if we could not tell.

**Domain 3: Reference Standard**

Risk of Bias: Could the reference standard, its conduct, or its interpretation have introduced bias?

• Signaling question 1: Is the reference standard likely to correctly classify the target condition?

NAAT is considered to be the gold standard for COVID. However, the accuracy of this reference standard is not 100%, especially late in the disease and it varies widely across the different non-respiratory samples. However, given that viral loads measured in NAATs correlate well with Antigen, we scored ‘yes’ for all studies included.

• Signaling question 2: Were the reference standard results interpreted without knowledge of the results of the index test?

We scored ‘yes’ if the test was performed immediately at the POC on fresh samples and ‘no’ if performed in the laboratory on stored samples unless blinding was specifically reported. We scored ‘unclear’ if we could not tell.

- Risk of Bias is scored ‘low concern’ if studies score ‘yes’ on all the question, ‘unclear concern’ if questions are answered with ‘yes’ and ‘unclear’, ‘intermediate concern’ if one question is answered with ‘no’, ‘high concern’ if two or more questions are answered with ‘no’.

Applicability: Are there concerns that the target condition as defined by the reference standard does not match the question? We judged applicability to be of ‘low concern’ for all studies.

**Domain 4: Flow and Timing**

Risk of Bias: Could the patient flow have introduced bias?

• Signaling question 1: Was there an appropriate interval between the index test and reference standard?

We expected specimens for Ag-RDT and the reference standards to be obtained at the same time and answered ’yes’ for all studies that meet these criteria. We answered ’no’ if specimens were collected for index and reference standard tests greater than 24h apart, and ’unclear’ if we could not tell.

• Signaling question 2: Did all patients receive the same reference standard?

Answer this question ‘yes’ if all studies used the same reference standard (acceptable reference standard as specified as a criterion for inclusion in the review), answer ‘no’ if different reference standards were used.

• Signaling question 3: Were all patients included in the analysis?

We determined the answer to this question by comparing the number of participants enrolled in the study with the number of participants included in the two-by-two tables. We answered ’yes’ if all participants enrolled in the study were tested with results presented and accounted for. We answered ’no’ if participants meeting enrolment criteria were not tested or results were not presented, and ’unclear’ if we could not tell.

- Risk of Bias is scored ‘low concern’ if studies score ‘yes’ on all the question, ‘unclear concern’ if questions are answered with ‘yes’ and ‘unclear’, ‘intermediate concern’ if one question is answered with ‘no’, ‘high concern’ if two or more questions are answered with ‘no’.

8. STATISTICAL ANALYSIS

We present estimates of sensitivity and specificity overall by using paired forest plots, and summarize results using average sensitivity and specificity in tables as appropriate. We estimate summary sensitivities and specificities with 95% confidence intervals (CI) using the bivariate model^13^, via the meqrlogit command of Stata/SE 16.0. Where studies presented only estimates of sensitivity or specificity, we will fit univariate random effects logistic regression models.

We will describe the studies according to the following characteristics

1. Test brand used
2. asymptomatic versus symptomatic
3. symptom duration prior to testing
4. Sampling (e.g. lay or professional). If lay, we will also consider supervised, non-supervised
5. Test conducted (e.g. lay or professional)
6. Type of RT-PCR
7. populations (e.g. pediatric versus adults)
8. use case (e.g. universal screening, targeted screening, diagnostic use case)
9. Setting (e.g. hospitals, schools, work environment)
10. viral load
11. Ct-value

For purpose of the WHO, we will perform a meta-analysis only on the self-testing studies (provided data allows). For the purpose of the publication, we will perform a meta-analysis combining self-testing and self-sampling studies and if data allows, we will consider subgroup meta-analyses by self-sampling/self-testing, test brand, symptom presence, professional versus self-sampling and testing, viral load (as defined by PCR). If meta-analysis is not possible in a subgroup, we will provide a descriptive overview, a range for accuracy and describe outliers. We will present ease of use (feasibility, usability, acceptability) in a descriptive matter as comparability of data is expected to be low (unless several studies use a standardized tool like the (modified) System Usability Scale^14,15^).

Investigations of heterogeneity

We examine heterogeneity between studies by visually inspecting the forest plots of sensitivity and specificity. Where adequate data is available for the subcategories, we investigated heterogeneity by including indicator variables in the random-effects logistic regression models. Absolute differences between the sensitivity or specificity and the P values are reported from the model.

Sensitivity analyses

We perform sensitivity analyses. Firstly, estimation of sensitivity and specificity if non-peer reviewed studies are excluded. Other sensitivity analyses will be considered depending on the study type.

Publication bias

Assessment if publication bias will be performed with funnel plots

References

1. World Health Organisation. Antigen-detection in the diagnosis of SARS-CoV-2 infection using rapid immunoassays Interim guidance, 11 September 2020. *World Heal Organ*. 2020;(September):1-9. https://apps.who.int/iris/handle/10665/334253.

2. Brümmer LE, Katzenschlager S, Gaeddert M, et al. *Accuracy of Novel Antigen Rapid Diagnostics for SARS-CoV-2: A Living Systematic Review and Meta-Analysis*. Vol 18.; 2021. doi:10.1371/journal.pmed.1003735

3. Dinnes J, Deeks J, Berhane S, et al. Rapid, point-of-care antigen and molecular-based tests for diagnosis of SARS-CoV-2 infection (Review). *Cochrane Database Syst Rev*. 2021. doi:10.1002/14651858.CD013705.pub2.

4. Lindner AK, Nikolai O, Rohardt C, et al. SARS-CoV-2 patient self-testing with an antigen-detecting rapid test: a head-to-head comparison with professional testing. *medRxiv*. 2021. https://doi.org/10.1101/2021.01.06.20249009.

5. Cockerill FR, Wohlgemuth JG, Radcliff J, et al. Evolution of Specimen Self-Collection in the COVID-19 Era: Implications for Population Health Management of Infectious Disease. *Popul Health Manag*. 2021;24(S1):S26-S34. doi:10.1089/pop.2020.0296

6. Therchilsen JH, von Buchwald C, Koch A, et al. Self-Collected versus Healthcare Worker-Collected Swabs in the Diagnosis of Severe Acute Respiratory Syndrome Coronavirus 2. *Diagnostics*. 2020;10(9):1-10. doi:10.3390/diagnostics10090678

7. Wachinger J, Schirmer M, Täuber N, McMahon SA, Denkinger CM. Experiences with opt-in, at-home screening for SARS-CoV-2 at a primary school in Germany: an implementation study. *BMJ Paediatr Open*. 2021;5(1):e001262. doi:10.1136/bmjpo-2021-001262

8. Wee LE, Conceicao EP, Sim JX-Y, Venkatachalam I, Wijaya L. Utilisation of SARS-CoV-2 rapid antigen assays in screening asymptomatic hospital visitors: mitigating the risk in low-incidence settings. *Int J Infect Dis*. 2021;114:132-134. doi:10.1016/j.ijid.2021.11.011

9. Tande AJ, Binnicker MJ, Ting HH, et al. SARS-CoV-2 Testing Before International Airline Travel, December 2020 to May 2021. *Mayo Clin Proc*. 2021;96(11):2856-2860. doi:10.1016/j.mayocp.2021.08.019

10. Young BC, Eyre DW, Kendrick S, et al. Daily testing for contacts of individuals with SARS-CoV-2 infection and attendance and SARS-CoV-2 transmission in English secondary schools and colleges: an open-label, cluster-randomised trial. *Lancet*. 2021;398(10307):1217-1229. doi:10.1016/S0140-6736(21)01908-5

11. Pollock NR, Savage TJ, Wardell H, et al. Correlation of SARS-CoV-2 nucleocapsid antigen and RNA concentrations in nasopharyngeal samples from children and adults using an ultrasensitive and quantitative antigen assay. *J Clin Microbiol*. 2021;59(4):1-10. doi:10.1101/2020.11.10.20227371

12. Whiting P, Rutjes AW, Reitsma JB, Bossuyt PM, Kleijnen J. The development of QUADAS: a tool for the quality assessment of studies of diagnostic accuracy included in systematic reviews. *BMC Med Res Methodol*. 2003. doi:10.1088/0004-637X/721/2/1919

13. Reitsma JB, Glas AS, Rutjes AWS, Scholten RJPM, Bossuyt PM, Zwinderman AH. Bivariate analysis of sensitivity and specificity produces informative summary measures in diagnostic reviews. *J Clin Epidemiol*. 2005;58(10):982-990. doi:10.1016/j.jclinepi.2005.02.022

14. Brooke J. SUS: A “Quick and Dirty” Usability Scale. In: *Usability Evaluation In Industry*. 1st ed. ; 1996:6.

15. Bangor A, Kortum PT, Miller JT. An empirical evaluation of the system usability scale. *Int J Hum Comput Interact*. 2008;24(6):574-594. doi:10.1080/10447310802205776

# Supplement Text 2 – Search Strategy

## Review questions

To assess the accuracy and ease-of-use of marketable antigen point of care diagnostics for SARS-CoV-2 compared to RT-PCR based on manufacturer independent evaluations.

Restriction: start Dezember 2019

## Definitions

P

| SARS-CoV-2 |
| --- |

I

| Antigen nachweisenden Schnelltests Ag RDT |
| --- |

## Strategie

| 1 | P |
| --- | --- |
| 2 | I |
| 3 | 1 AND 2 |

## Searched Databases

- PubMed
- Web of Science Core Collection
- BioRxiv
- MedRxiv

## PubMed

P

| (**"Severe Acute Respiratory Syndrome Coronavirus 2"[Mesh] OR**  **"COVID-19" [Mesh] OR**  **"Betacoronavirus"[Mesh] OR**  **"Coronavirus"[Mesh] OR**  covid*[tw] OR  "coronavirus*"[tw] OR  "corona virus*"[tw] OR  ncov*[tw] OR  "n cov*"[tw] OR  sarscov*[tw] OR  "sars cov*"[tw] OR  "2019nCoV*"[tw] OR  "2019 nCoV*"[tw] OR  "sars2*"[tw] OR  "sars 2*"[tw]) |
| --- |

I

| **"Point-of-Care Testing"[Mesh] OR**  Antigen[tw] OR  “Lateral flow”[tw] OR  RDT[tw] OR  (("Point of Care*"[tw] OR  "Bedside*"[tw] OR  Rapid*[tw])  AND  Test*[tw]) |
| --- |

**P**

**1 (99976)**

("Severe Acute Respiratory Syndrome Coronavirus 2"[Supplementary Concept] OR "COVID-19"[Supplementary Concept] OR "Betacoronavirus"[MeSH Terms] OR "Coronavirus"[MeSH Terms] OR "covid*"[Text Word] OR "coronavirus*"[Text Word] OR "corona virus*"[Text Word] OR "ncov*"[Text Word] OR "n cov*"[Text Word] OR "sarscov*"[Text Word] OR "sars cov*"[Text Word] OR "2019ncov*"[Text Word] OR "2019 ncov*"[Text Word] OR "sars2*"[Text Word] OR "sars 2*"[Text Word])

**I**

**2 (816722)**

("Point-of-Care Testing"[MeSH Terms] OR "antigen"[Text Word] OR "lateral flow"[Text Word] OR "RDT"[Text Word] OR (("point of care*"[Text Word] OR "bedside*"[Text Word] OR "rapid*"[Text Word]) AND "test*"[Text Word]))

**3 (1637239)**

2019/12/01:2021/11/19[Date - Publication]

**1 AND 2 AND 3 (2990)**

## Web of Science Core Collection

P

| "covid*" OR  "coronavirus*" OR  "corona virus*" OR  "ncov*" OR  "n cov*" OR  "sarscov*" OR  "sars cov*" OR  "2019nCoV*" OR  "2019 nCoV*" OR  "sars2*" OR  "sars 2*" |
| --- |

I

| "antigen" OR  "Lateral flow" OR  "RDT" OR  (("Point of Care*" OR  "Bedside*" OR  "Rapid*")  AND  Test*)) |
| --- |

P

**1 (56968)**

TS=("covid*" OR "coronavirus*" OR "corona virus*" OR "ncov*" OR "n cov*" OR "sarscov*" OR "sars cov*" OR "2019nCoV*" OR "2019 nCoV*" OR "sars2*" OR "sars 2*")

I

**2 (34058)**

TS=("antigen" OR "Lateral flow" OR "RDT" OR (("Point of Care*" OR "Bedside*" OR "Rapid*")AND Test*))

**1 AND 2 (1568)**

Filter (year to date 2022/11/07)

## Bio_MedRxiv

https://europepmc.org/

P

| Covid* OR  Coronavirus* OR  "corona virus*" OR  Ncov* OR  "n cov*" OR  Sarscov* OR  "Sars cov*" OR  2019nCoV* OR  "2019 nCoV*" OR  sars2* OR  "sars 2*" |
| --- |

I

| "Antigen*" OR  "Lateral flow*" OR  "RDT" OR  "Rapid test*" OR  "Bedside*" OR  "Point of Care*" |
| --- |

**P AND I (2225)**

(covid* OR Coronavirus* OR "corona virus*" OR ncov* OR "n cov*" OR sarscov* OR "sars cov*" OR 2019nCov* OR "2019 nCov*" OR sars2* OR "sars 2*")

AND

(Antigen OR "Lateral flow" OR RDT OR "Rapid test*" OR Bedside* OR "Point of Care*")

AND

(PUBLISHER:MedRxiv OR PUBLISHER:BioRxiv)

AND

FIRST_PDATE:[2019-12-01 TO 2022-11-07]

# Supplement Text 3 – QUADAS assessment interpretation guide

**Domain 1 Patient Selection:**

Risk of Bias: Could the selection of patients have introduced bias?

• Signaling question 1: Was a consecutive or random sample of patients or specimens enrolled?

We scored ‘yes’ if the study enrolled a consecutive or random sample of eligible patients; ‘no’ if the study selected patients by convenience, and ‘unclear’ if the study did not report the manner of patient selection or unable to tell.

• Signaling question 2: Was a case-control design avoided?

We scored ‘no’ if the study selected samples with a known rt-PCR results. We scored ‘yes’ if the status of the samples was unknown. We scored ‘unclear’ if we could not tell.

• Signaling question 3: Did the study avoid inappropriate exclusions?

We scored ’yes’ to studies which included all participants regardless of symptoms or duration of symptoms. We scored ’no’ if studies excluded participants on the basis of symptoms or duration of symptoms. We scored ’unclear’ if we could not tell.

We considered any studies that included patients based on a previous positive rt-PCR results as monitoring studies and thus judged the applicability of the study population to be of ‘high concern’ (see below).

- Risk of Bias was scored ‘low concern’ if studies score ‘yes’ on all the question, ‘unclear concern’ if questions are answered with ‘yes’ and ‘unclear’, ‘intermediate concern’ if one question is answered with ‘no’, ‘high concern’ if two or more questions are answered with ‘no’.

Applicability: Are there concerns that the included patients and setting do not match the review question?

We were interested in how Ag-RDT performs in patients whose specimens were evaluated as they would be in routine practice. We scored ’low concern’ if the study was conducted in a routine practice setting. We scored ‘high concern’ if Ag-RDT were evaluated for end of quarantine evaluation or monitoring. We scored ‘unclear’ if we could not tell.

**Domain 2: Index Test**

Risk of Bias: Could the conduct or interpretation of the index test have introduced bias?

• Signaling question 1: Were the index test results interpreted with knowledge of the results of the reference standard?

We answered ’yes’ if the study interpreted the result of Ag-RDT blinded to the result of the reference standard or for studies in which Ag-RDT was performed on fresh specimens, since reference standard results would be unavailable at the time of test interpretation. We answered ’no’ if the study did not interpret the result of Ag-RDT blinded to the result of the reference standard. We answered ’unclear’ if stored specimens were tested or we could not tell if the index test results were interpreted without knowledge of the reference standard results.

• Signaling question 2: If a threshold was used, was it prespecified?

We answered ’yes’ if the threshold was prespecified or if the tests was performed by IFU. We scored ’no’ if the threshold was not prespecified, and ’unclear’ if we could not determine if the threshold was prespecified or not.

- Risk of Bias was scored ‘low concern’ if studies score ‘yes’ on all the question, ‘unclear concern’ if questions are answered with ‘yes’ and ‘unclear’, ‘intermediate concern’ if one question is answered with ‘no’, ‘high concern’ if two or more questions are answered with ‘no’.

Applicability: Are there concerns that the index test, its conduct, or its interpretation differ from the review question? If index test methods vary from those specified in the review question, concerns about applicability may exist. We judged ’high concern’ if the test procedure was inconsistent with the manufacturer recommendations, ’low concern’ if the test procedure was consistent with the manufacturer recommendations, and ’unclear concern’ if we could not tell.

**Domain 3: Reference Standard**

Risk of Bias: Could the reference standard, its conduct, or its interpretation have introduced bias?

• Signaling question 1: Is the reference standard likely to correctly classify the target condition?

Viral culture is considered the gold standard for SARS-CoV-2 detection. Since viral culture is available in research settings only, NAAT is the considered routine standard for SARS-CoV-2 testing. However, the accuracy of this reference standard is not 100%, especially late in the disease, where it varies widely across the different non-respiratory samples and may detect non-viable virus Still, given that viral loads measured in NAATs correlate well with Antigen, we scored ‘yes’ for all studies using a NAAT as reference standard.

• Signaling question 2: Were the reference standard results interpreted without knowledge of the results of the index test?

We scored ‘yes’ if the test was performed ahead of the Ag-RDT or blinding was specifically reported. We scored ‘unclear’ if we could not tell.

- Risk of Bias is scored ‘low concern’ if studies score ‘yes’ on all the question, ‘unclear concern’ if questions are answered with ‘yes’ and ‘unclear’, ‘intermediate concern’ if one question is answered with ‘no’, ‘high concern’ if two or more questions are answered with ‘no’.

Applicability: Are there concerns that the target condition as defined by the reference standard does not match the question?

We judged applicability to be of ‘low concern’ for all studies.

**Domain 4: Flow and Timing**

Risk of Bias: Could the patient flow have introduced bias?

• Signaling question 1: Was there an appropriate interval between the index test and reference standard?

We expected specimens for Ag-RDT and the reference standards to be obtained at the same time and answered ’yes’ for all studies that meet these criteria. We answered ‘unclear’ if we could not tell.

• Signaling question 2: Did all patients receive the same reference standard?

We answered this question ‘yes’ for all studies that used the same rt-PCR for all samples and ‘no’ if the samples were analyzed by different types of rt-PCR. We scored ‘unclear’ if we could not tell the used rt-PCR.

• Signaling question 3: Were all patients included in the analysis?

We determined the answer to this question by comparing the stated population size with the number of samples included in the two-by-two tables. We answered ’yes’ if the whole population was included in the analysis or any excluded samples were reasoned for. We answered ‘no’ if samples were excluded without a given reason. We answered ‘unclear’ if we could not tell.

- Risk of Bias is scored ‘low concern’ if studies score ‘yes’ on all the question, ‘unclear concern’ if questions are answered with ‘yes’ and ‘unclear’, ‘intermediate concern’ if one question is answered with ‘no’, ‘high concern’ if two or more questions are answered with ‘no’.

# Supplement Figure 1 – QUADAS


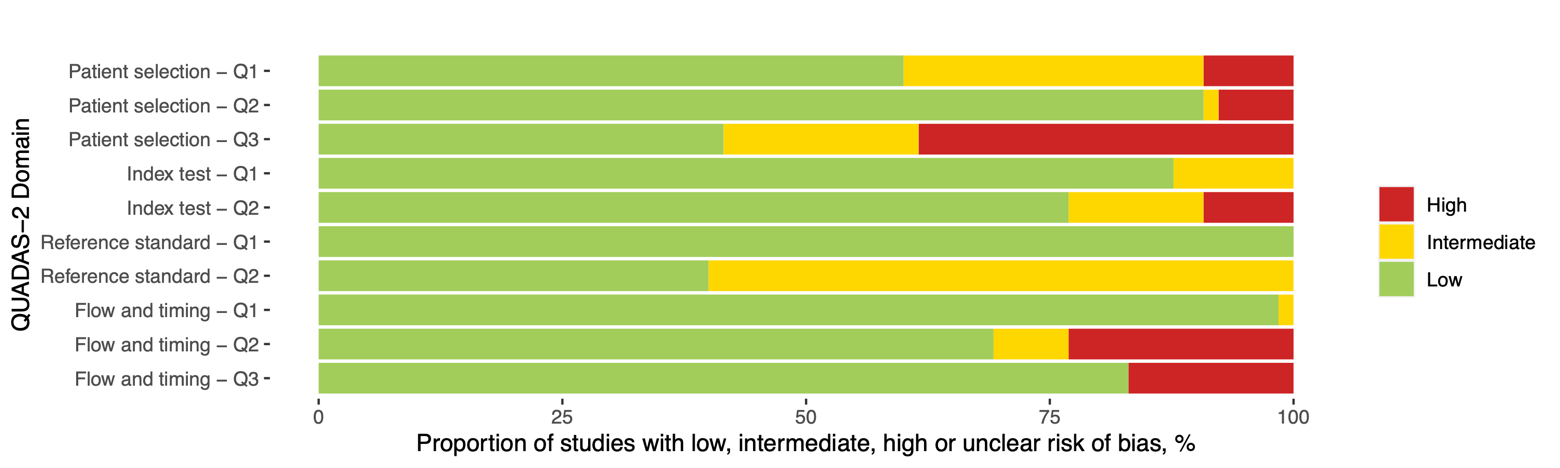


# Supplement Figure 2 – Funnel Plot


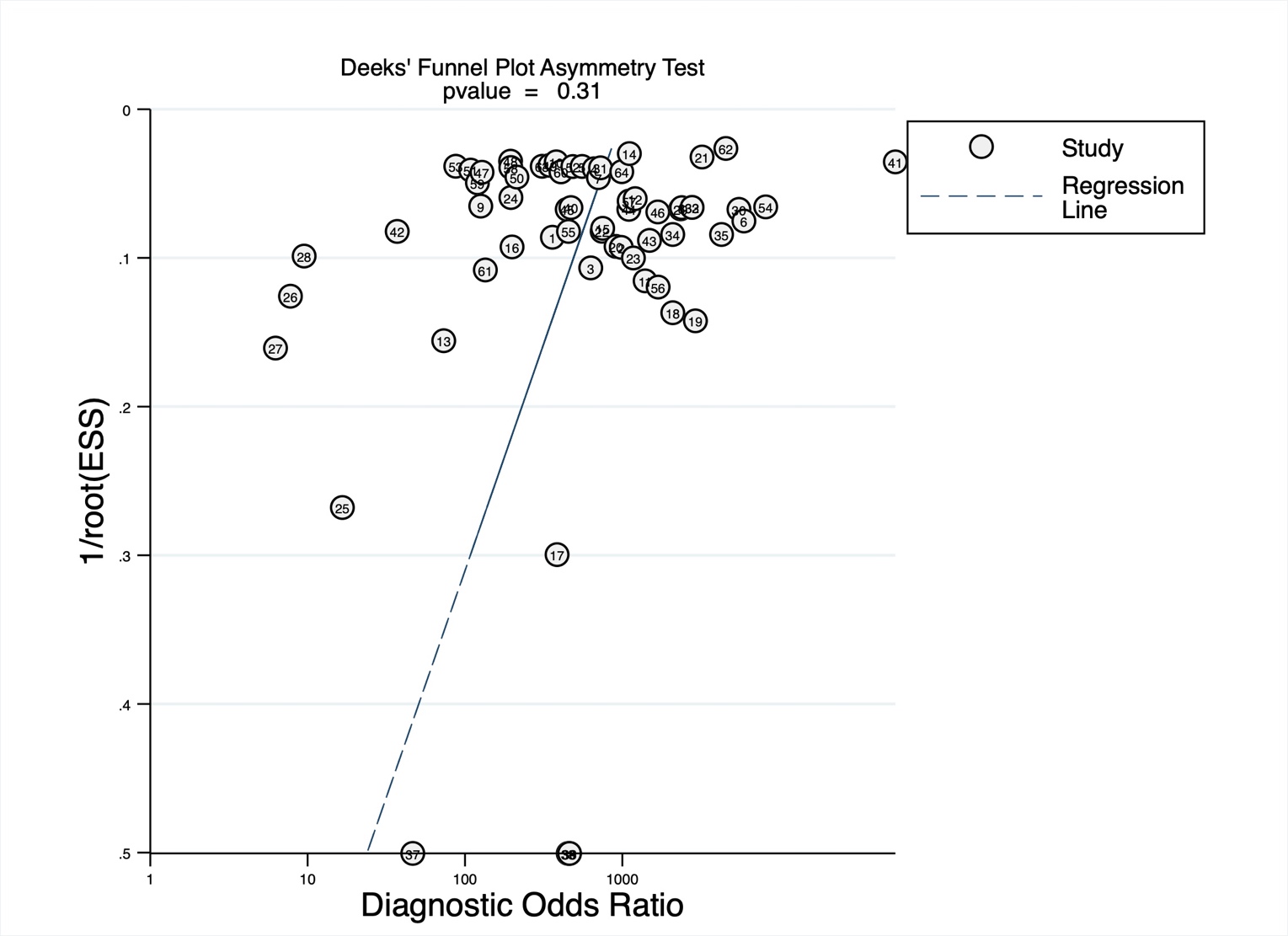


# Supplement Figure 3 – Pooled Cohen’s kappa

Pooled concordance from self-sampling versus professional Ag-RDTs (both sampling and testing performed by professional); Abbreviations: a = self-test & professional test positive; b = self-test positive & professional test negative; c = self-test negative & professional test positive; d = self-test & professional test negative; CI = confidence interval

# Supplement Figure 4 – Accuracy for self-testing studies according to IFU-conformity

Abbreviations: CI = confidence interval; IFU = instructions for use.

# Supplement Figure 5 – Accuracy for self-sampled studies according to IFU-conformity

Abbreviations: CI = confidence interval; IFU = instructions for use.

Supplement Figure 6 – Accuracy for high income countries

Abbreviations: CI = confidence interval.

# Supplement Figure 7 – Accuracy for middle income countries

Abbreviations: CI = confidence interval.

# Supplement Figure 8 – Sensitivity analysis excluding case-control studies

Abbreviations: CI = confidence interval.

# Supplement Figure 9 – Sensitivity analysis excluding manufacturer-dependent studies

Abbreviations: CI = confidence interval.

å

# Supplement Figure 10 – Sensitivity analysis excluding preprints

Abbreviations: CI = confidence interval
