# Supplementary material for: Comparing SARS-CoV-2 antigen-detection rapid diagnostic tests for COVID-19 self-testing/self-sampling with molecular and professional-use tests: a systematic review and meta-analysis
Source: Sci Rep. 2023 Dec 11;13:21913. doi: 10.1038/s41598-023-48892-x (PMC10713601; doi:10.1038/s41598-023-48892-x)
Supplement: Supplementary file 4 — Supplementary Table 3. [file 41598_2023_48892_MOESM4_ESM.docx]

|  | **ST/SS vs. professional-testing** | | | | **ST/SS vs. RT-PCR** | | | | **Professional-testing vs. RT-PCR** | | | | **OPA (%)** | **PPA (%)** | **NPA (%)** |
| --- | --- | --- | --- | --- | --- | --- | --- | --- | --- | --- | --- | --- | --- | --- | --- |
| **Study** | **a** | **b** | **c** | **d** | **a** | **b** | **c** | **d** | **a** | **b** | **c** | **d** |  |  |  |
| Lindner 2020 | 29 | 2 | 3 | 255 | 29 | 2 | 10 | 248 | 31 | 1 | 8 | 249 | 98.27 | 90.62 | 99.22 |
| Chiu 2021 | 44 | 2 | 4 | 247 | 62 | 10 | 13 | 264 | 64 | 14 | 11 | 260 | 97.98 | 91.67 | 99.19 |
| Klein 2021 | 37 | 3 | 5 | 245 | 38 | 2 | 6 | 235 | 40 | 2 | 4 | 235 | 97.24 | 88.1 | 98.79 |
| Nikolai 2021 | 30 | 2 | 1 | 63 | 31 | 1 | 3 | 61 | 31 | 0 | 5 | 96 | 96.88 | 96.77 | 96.92 |
| Lindner 2021 | 33 | 1 | 3 | 108 | 33 | 0 | 7 | 104 | 34 | 1 | 6 | 105 | 97.24 | 91.67 | 99.08 |
| Chen NP 2022 | 17 | 2 | 0 | 11 | 19 | 0 | 8 | 3 | 17 | 0 | 3 | 10 | 93.33 | 100 | 84.61 |
| Chen OP 2022 | 41 | 3 | 1 | 23 | 35 | 9 | 8 | 16 | 36 | 6 | 7 | 19 | 94.12 | 97.62 | 88.46 |

Supplement Table 3 – Accuracy of all head-to-head studies assessing self-testing or self-sampling Ag-RDT, professional-testing Ag-RDT, and RT-PCR

Abbreviation: Abbreviations: a = self-test & professional test positive; b = self-test positive & professional test negative; c = self-test negative & professional test positive; d = self-test & professional test negative; RT-PCR = Reverse transcription polymerase chain reaction; ST = self-testing; SS = self-sampling; OPA = overall percentage agreement; PPA = positive percentage agreement; NPA = negative percentage agreement.
